# *STAT1* and *IL-7* as potential diagnostic biomarkers for distinguishing high-grade from low-grade serous ovarian cancer: a multi-cohort analysis

**DOI:** 10.3389/fimmu.2026.1779912

**Published:** 2026-04-14

**Authors:** Zhuna Wu, Xuanxuan Zhuang, Xinxin Zhan, Weihong Chen, Yajing Xie, Zhimei Zhou, Li Huang, Liying Sheng, Yueli Wang, Binbin Chen, Ruiyun Wu, Yumin Ke

**Affiliations:** 1 Department of Gynecology and Obstetrics, The Second Affiliated Hospital of Fujian Medical University, Quanzhou, Fujian, China; 2 Department of Reproductive Medicine, The Second Affiliated Hospital of Fujian Medical University, Quanzhou, Fujian, China

**Keywords:** diagnostic immune biomarkers, high-grade serous ovarian cancer (HGSOC), IL-7, low-grade serous ovarian cancer (LGSOC), STAT1

## Abstract

**Background:**

High-Grade Serous Ovarian Carcinoma (HGSOC) and Low-Grade Serous Ovarian Carcinoma (LGSOC) are distinct subtypes of epithelial ovarian cancer with significant differences in pathogenesis and prognosis, posing challenges for precise diagnosis. Identifying reliable biomarkers is crucial for improving differential diagnosis and clinical management.

**Methods:**

Transcriptome RNA-seq data of HGSOC and LGSOC were obtained from the GEO database (GSE27651, GSE126132). Differentially expressed immune-related genes (DIRGs) were identified. Functional enrichment analysis and protein-protein interaction (PPI) network construction were performed. The Least Absolute Shrinkage and Selection Operator (LASSO) regression and multiple Support Vector Machine Recursive Feature Elimination (mSVM-RFE) algorithms were used to select predictive genes. Diagnostic performance was evaluated using receiver operating characteristic (ROC) curves, and a nomogram was developed. Findings were validated in an independent dataset and via immunohistochemistry (IHC). The CIBERSORT algorithm assessed correlations between key DIRGs and tumor-infiltrating immune cells, with false discovery rate (FDR) correction applied for multiple testing.

**Results:**

Seventy-one DIRGs were identified in HGSOC versus LGSOC, predominantly enriched in cytokine-mediated signaling, cytokine-cytokine receptor interaction, and JAK-STAT pathways. *STAT1* and *IL-7* were selected as diagnostic biomarkers, with area under the curve (AUC) values of 0.908 and 0.842 in the train group. Respectively, validation in an independent merged cohort (GSE14001, GSE73168, GSE146965; 55 HGSOC, 13 LGSOC) yielded AUCs of 0.703 (95% CI: 0.517–0.889) for *STAT1* and 0.706 (95% CI: 0.501–0.912) for *IL-7*. IHC confirmed significantly higher *STAT1* and lower *IL-7* protein expression in HGSOC tissues (P < 0.05). Immune microenvironment analysis revealed that HGSOC exhibited significantly higher fractions of naïve B cells, M2 macrophages, and neutrophils, and lower fractions of resting memory CD4+ T cells and eosinophils after FDR correction (all q < 0.05). *STAT1* expression was strongly positively correlated with M1 macrophages (ρ = 0.688, q = 9.9×10^-^^8^), and showed correlation trends with other immune cell types that did not remain significant after FDR correction. *IL-7* expression exhibited a negative correlation trend with neutrophils (ρ = –0.372, raw P = 0.0048, q = 0.100).

**Conclusion:**

*STAT1* and *IL-7* are consistently differentially expressed between HGSOC and LGSOC and may serve as ancillary diagnostic biomarkers in histologically ambiguous cases. However, their clinical utility—particularly in multi-gene combinations—requires prospective validation.

## Introduction

Ovarian malignancy is a common malignant tumor of the female reproductive system, with a mortality rate second only to cervical cancer and endometrial cancer (1). Over 90% of ovarian cancers originate from ovarian epithelial cells (2). Based on morphological differences in epithelial cells, epithelial ovarian cancer is mainly classified into serous carcinoma (approximately 71%), clear cell carcinoma (12%), endometrioid carcinoma (11%), and mucinous carcinoma (3%) (2). Among these, serous carcinoma is highly aggressive and is often diagnosed at an advanced stage (51% at stage III and 29% at stage IV), leading to poor prognosis in patients (3, 4). With advances in molecular biology, ovarian serous carcinoma can be further divided into low-grade (LGSOC, type I) and high-grade (HGSOC, type II), which show significant differences in molecular characteristics and clinical outcomes (5). LGSOC is more common in younger women, has a better prognosis but is relatively chemotherapy-resistant, and often progresses from borderline tumors (6, 7). In contrast, HGSOC mostly originates from the fallopian tube epithelium and progresses rapidly (8). Accurate and early diagnosis is crucial for the treatment and prognosis of ovarian cancer patients.

HGSOC exhibits a complex tumor microenvironment (TME) enriched with CD3^+^ T cells and CD163^+^ tumor-associated macrophages (TAMs), associated with better prognosis and platinum sensitivity (9, 10). Its immune landscape is categorized into five subtypes linked to clinical outcomes (10). Notably, the high prevalence of *TP53* mutations (>90%) may promote immune evasion by altering immunogenicity or antigen presentation (11, 12), while peritoneal metastasis and ascites can further suppress local immunity (13). LGSOC, in contrast, demonstrates a distinct TME shaped by its molecular profile (e.g., frequent *KRAS* and rare *TP53* mutations), which likely influences immune cell recruitment and function (11, 14, 15). Typically exhibiting low immunogenicity and sparse immune infiltration, LGSOC shows limited response to immunotherapy (11, 15). Its immune escape may stem from low mutational burden and scarce neoantigen generation, hindering T-cell activity (11, 15). The immune mechanisms underlying its progression from serous borderline tumors remain unclear (14, 16).

To improve the prognostic results for patients with HGSOC, it is essential to explore the cellular and molecular differences between HGSOC and LGSOC so as to formulate more effective treatment strategies customized for HGSOC.

## Materials and methods

### Collection and processing of data

Gene expression profiles of HGSOC and LGSOC were obtained from GEO (https://www.ncbi.nlm.nih.gov/gds) (all datasets are microarray-based; platforms detailed in **Table 1**). For each dataset, series matrix files were downloaded, probes were mapped to gene symbols using platform annotation files, and multiple probes per gene were collapsed to the mean expression value (avereps, limma). Preprocessing was performed separately for the training (GSE27651, GSE126132) and validation (GSE14001, GSE73168, GSE146965) cohorts. Within each cohort, data were log2-transformed if not already on a log scale, quantile-normalized (normalizeBetweenArrays, limma), and the common genes across datasets were retained. Batch effects were then corrected using ComBat (sva package) independently for the training and validation sets to prevent data leakage. To assess batch effect removal, we performed principal component analysis (PCA) before and after correction for each cohort, and also on the combined training and test sets after separate correction (**Supplementary Figure S1**). The proportion of variance explained by batch (or cohort) was quantified using permutational multivariate analysis of variance (PERMANOVA, adonis2 with 999 permutations); results are summarized in **Supplementary Table S1**. Additionally, relative log expression (RLE) plots were generated to visualize expression deviations (**Supplementary Figure S2**). To further illustrate the impact of batch correction, we generated boxplots of the median expression levels per sample across datasets before and after ComBat correction (**Supplementary Figure S3**). Summary statistics for each dataset and correction stage are provided in **Supplementary Table S2**.

**Table 1:** Summary of GEO datasets used in this study.

Data set ID	platform	LGSOC	HGSOC
Train group			
GSE27651	GPL570 (Affymetrix U133 Plus 2.0)	13	22
GSE126132^a^	GPL10558 (Illumina HT−12 V4.0)	0	34
Test group			
GSE14001	GPL570 (Affymetrix U133 Plus 2.0)	10	10
GSE73168	GPL570 (Affymetrix U133 Plus 2.0)	3	5
GSE146965^b^	GPL23126 (Affymetrix Clariom D)	0	40

^a^GSE126132 contains exclusively HGSOC samples. It was merged with GSE27651 to form the training cohort and was used in all training−phase analyses, including differential expression (DEG) screening, batch effect correction, immune infiltration deconvolution, and co−expression network construction.

^b^GSE146965 contains exclusively HGSOC samples. It was merged with GSE14001 and GSE73168 for the combined validation ROC analysis (**Figure 6F**) and was also used for expression trend confirmation. It was excluded from independent ROC−based diagnostic performance evaluation due to the absence of LGSOC controls.

It should be noted that GSE126132 (training cohort) and GSE146965 (validation cohort) contain only HGSOC samples. GSE126132 was merged with GSE27651 for all training-set analyses, including differential expression screening and machine learning. GSE146965 was merged with GSE14001 and GSE73168 to form the combined validation set for ROC analysis; separate ROC results for datasets containing both subtypes are provided in the **Supplementary Material**.

Differentially expressed genes (DEGs) between HGSOC and LGSOC were identified using limma with criteria |log_2_FC| ≥ 1 and adjusted P < 0.05 (Benjamini–Hochberg). Immune-related genes (IRGs) were downloaded from ImmPort (https://www.immport.org/shared/) (**Supplementary Table S3**). Intersection of DEGs and IRGs yielded 71 differentially expressed immune-related genes (DIRGs) for subsequent analyses. All preprocessing and quality control were performed in R (v4.1.3) using the limma, sva, vegan, and ggplot2 packages.

### Functional enrichment analyses

We carried out functional annotation analysis via GO and KEGG pathway enrichment using the “clusterProfiler”, “org.Hs.eg.db”, “enrichplot”, and “DOSE” packages. We systematically characterized the enriched terms in three GO domains: biological processes (BP), molecular functions (MF), and cellular components (CC), as well as the curated KEGG signaling pathways. We visualized the enrichment results with dot plots created by the “ggplot2” package, which adopted a modular visualization framework. To account for multiple testing, p-values were adjusted using the Benjamini–Hochberg false discovery rate (FDR) procedure. Terms with raw p < 0.05 and adjusted p (q) < 0.05 were considered significantly enriched.

### Building and analyzing the protein - protein interaction network

We used the STRING website (https://string-db.org/) to look for a PPI network. In the “multiple proteins” module, we input 71 DIRGs, and in the organism module, we selected “Homo sapiens”. Gene symbols were retrieved from protein IDs, and PPIs lacking associated gene names were removed. After that, we made use of Cytoscape 3.10.0 to build the PPI network. The cytoHubba plugin was used to help identify hub genes.

### Identification of hub DIRGs

To identify the most central DIRGs potentially involved in HGSOC pathogenesis, we first constructed a PPI network of the 71 DIRGs using the STRING database. The interaction data were imported into Cytoscape software, and the cytoHubba plugin was employed to rank genes based on the Maximal Clique Centrality (MCC) algorithm. The top 10 genes with the highest MCC scores were designated as hub DIRGs. Subsequently, to explore the interrelationships among these hub genes, we performed pairwise Spearman correlation analysis on their expression levels in the training cohort. Correlation coefficients and statistical significance were calculated, and a co-expression network was visualized to reveal potential regulatory interactions among the hub DIRGs.

### Construction of a predictive model for HGSOC diagnosis based on DIRGs

To further refine the set of hub DIRGs and identify the most robust diagnostic biomarkers for distinguishing HGSOC from LGSOC, we applied two complementary machine learning algorithms: LASSO regression and SVM-RFE. These analyses were performed on the expression data of the 10 hub DIRGs in the training cohort. For LASSO regression, we utilized the glmnet package in R. The model was fit using binomial logistic regression with a single run of 10-fold cross-validation. The optimal penalization parameter λ was chosen as lambda.min (the value minimizing the mean cross-validated deviance), because our primary aim was to select a comprehensive set of features for further validation, and the selected genes were subsequently tested in independent cohorts to control for overfitting. Genes with non-zero coefficients at the optimal λ were retained. For SVM-RFE, we employed a linear kernel function with default cost parameters (C = 1). The algorithm was applied with a single run of 10-fold cross-validation to recursively eliminate features and rank them by their importance. The optimal number of features was selected based on the point of lowest cross-validation error. Finally, the genes selected by both LASSO and SVM-RFE were considered as the most promising diagnostic biomarkers for HGSOC.

We used the R package “pROC” to draw ROC curves for the dataset. The area under the curve (AUC) was calculated to evaluate the ability of the potential biomarker to differentiate HGSOC from LGSOC tissue.

Learning curve analysis was performed to assess overfitting of the four-gene logistic regression model. Using the training cohort (n=69), we iteratively increased the training set size from 30% to 100% in 10% increments, with 10 repetitions of 5-fold cross-validation at each step. The mean training and cross-validation AUCs were plotted against training sample size to visualize the convergence gap.

### Develop and verify the nomogram model regarding the diagnostic capacity for HGSOC

We constructed a nomogram model using the “rms” and “rmda” packages to forecast the diagnosis of HGSOC. A logistic regression model was built using the two candidate genes (*STAT1* and *IL-7*) as predictors. These two genes were selected from an initial panel of four genes (*STAT1, TNFRSF1A, EGFR, IL-7*) identified by LASSO and SVM-RFE, after *TNFRSF1A* and *EGFR* demonstrated inconsistent differential expression in external validation cohorts (GSE14001, GSE73168, GSE146965).

The predictive performance was evaluated using the area under the AUC with 95% confidence intervals calculated by the Delong test (pROC package). The optimal cutoff threshold was determined by the maximum Youden index (sensitivity + specificity - 1) from the ROC curve. Model calibration was assessed using the Brier score and expected calibration error (ECE). Calibration curves were constructed with bootstrap resampling (1,000 replicates) using the calibrate function to correct for overfitting bias.

Decision curve analysis (DCA) was performed using the rmda package to evaluate the net benefit of the model across different threshold probabilities (bootstraps = 50). The score of each factor in the nomogram is denoted as “points,” and the cumulative score of all factors is called “total points.” The calibration curves were generated to evaluate the predictive performance of the nomogram model, with predicted probabilities plotted against observed outcomes.

To assess the robustness of the selected biomarkers, we performed bootstrap stability analysis with 1,000 iterations using 80% stratified sampling. Additionally, we performed independent replication of the ‘difference analysis-screening-evaluation’ pipeline within each training GSE dataset where possible. Due to the dataset composition (GSE126132 contains only HGSOC samples without LGSOC controls), standard leave-one-dataset-out cross-validation could not be performed.

### The immune cell infiltration in HGSOC and its biomarkers

We used the CIBERSORT algorithm (http://cibersort.stanford.edu/) with the LM22 signature matrix, which contains 547 genes distinguishing 22 immune cell subtypes, to quantify the relative abundances of infiltrating immune cells in HGSOC and LGSOC samples. The normalized gene expression matrix was used as input. To ensure the reliability of deconvolution results, we performed 1,000 permutations for each sample to obtain empirical p-values, and only samples with CIBERSORT output p < 0.05 were retained for downstream correlation analyses. Prior to CIBERSORT analysis, batch effects across datasets had been corrected using the ComBat algorithm, as detailed in the data preprocessing section.

To validate the robustness of the immune infiltration estimates obtained by CIBERSORT, we additionally employed the xCell algorithm (https://github.com/dviraran/xCell) using the same normalized expression matrix. xCell was run with default parameters, and only immune cell types that overlapped with those estimated by CIBERSORT were retained for subsequent correlation analysis. The results from both algorithms were compared to assess consistency.

### Patient and tissue samples

Twelve paraffin - embedded specimens from patients with LGSOC and 26 from patients with HGSOC, who had undergone ovarian cystectomy or oophorectomy at the Second Affiliated Hospital of Fujian Medical University between November 2015 and December 2024, were collected. The Research Ethics Committee of the Second Affiliated Hospital of Fujian Medical University approved this study before its initiation.

### Immunohistochemistry

IHC staining was performed following the previously described procedures. We used primary antibodies, including anti - STAT1 (Affbiotech, China) and anti - IL-7 (Affbiotech, China). The staining intensity of *STAT1* and *IL-7* was categorized into four levels: negative (scored as 0), light yellow (1 point), brownish yellow (2 points), and tan (3 points). For the number of stained cells, the evaluation was based on the following ratios: if less than one - third, 1 point was given; if between one - third and two - thirds, 2 points; if more than two - thirds, 3 points. The final expression scores of *STAT1* and *IL-7* were calculated by multiplying the intensity score and the percentage score. To determine the optimal cutoff for distinguishing HGSOC from LGSOC, ROC curve analysis was performed on the IHC scores, and the Youden index (sensitivity + specificity – 1) was used to select the threshold that maximized diagnostic accuracy (see **Supplementary Figure S4**; **Supplementary Table S4**). Based on the ROC results, samples with a total score ≥4 for *STAT1* and ≥5 for *IL-7* were defined as high expression. All immunohistochemically stained sections were scored independently by two pathologists specializing in gynecologic oncology, who remained blinded to the clinical diagnoses and group assignments. Each pathologist separately assessed the intensity of staining and the proportion of positive cells, and these two measures were multiplied to obtain an overall score. Whenever the initial scores diverged by more than 30%, the two pathologists re−examined the slides together using a multi−headed microscope and reached a consensus final score for each specimen.

#### Statistical analysis

Main analyses were performed in R (v4.1.3), while additional validation analyses were conducted in R (v4.3.3). Details of all R packages and versions are provided in **Supplementary Table S5**. For immune cell proportion comparisons between HGSOC and LGSOC, the two-tailed Mann–Whitney U test was applied; correlations between gene expression (*STAT1* and *IL-7*) and immune cell fractions were evaluated using Spearman’s rank correlation. To account for multiple testing, raw p−values from these analyses were adjusted using the Benjamini–Hochberg FDR procedure, with FDR q < 0.05 considered statistically significant. Differential expression analysis was conducted using the limma package (|log_2_FC| ≥ 1, adjusted P < 0.05). Feature selection employed LASSO regression and SVM−RFE. Diagnostic performance was assessed by ROC curve analysis; All ROC curves were annotated with the sample size, the AUC with its 95% confidence interval (calculated by bootstrap resampling), and the optimal threshold point (Youden index) from the training set. The same threshold was applied to independent external validation datasets. Statistical significance was defined as raw P < 0.05, with FDR correction applied where indicated.

## Results

### Research process

**Figure 1:**
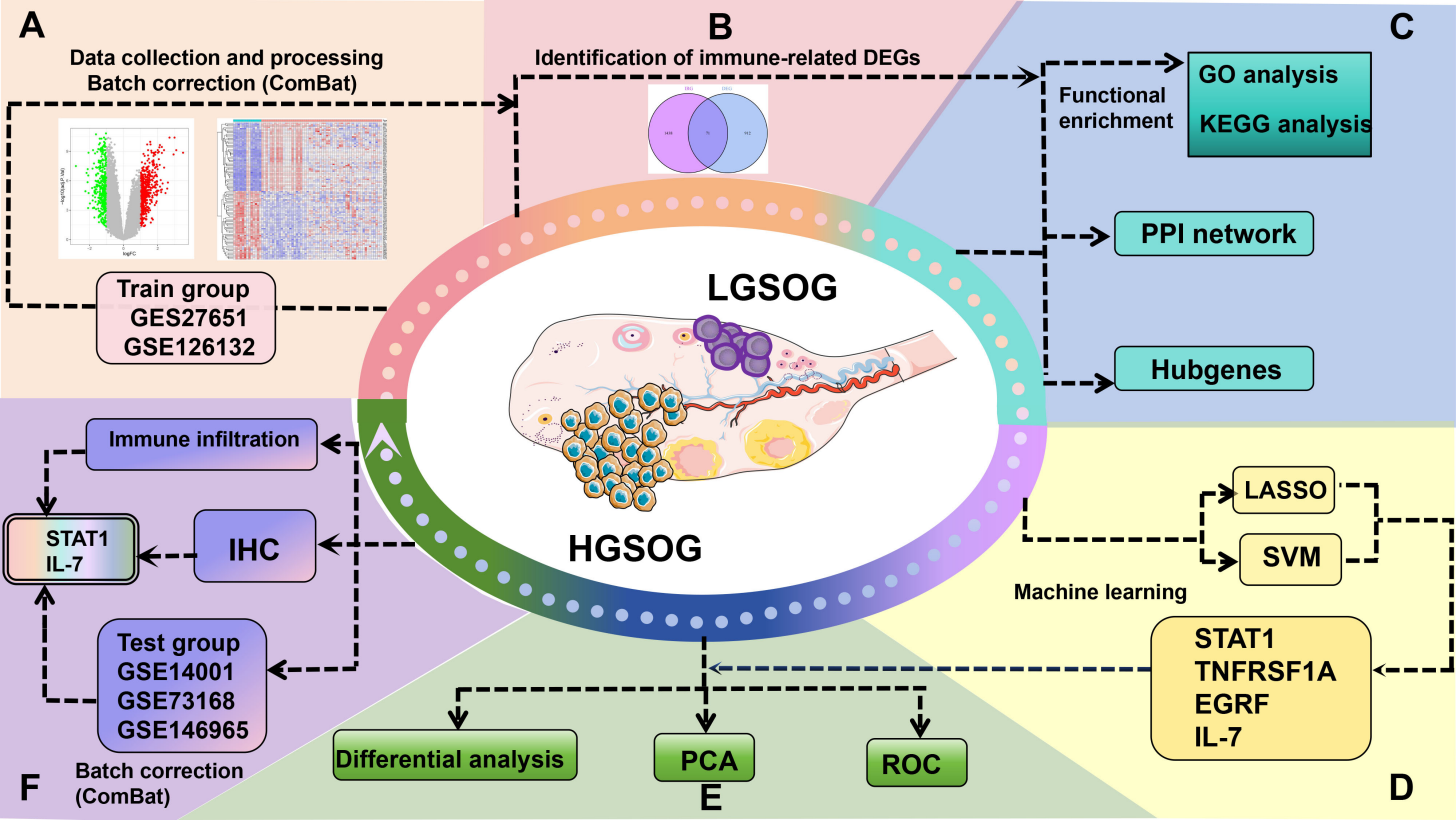
Confirmed that the schematic accurately reflects microarray-based analysis.

In this study, we adopted the analytical procedure depicted in **Figure 1**. Transcriptome RNA-seq data were retrieved from the GEO database. By combining DEGs and IRGs, we screened for the overlapping DIRGs between the LGSOC and HGSOC groups. Subsequently, GO, KEGG, PPI, and hub gene network analyses were carried out on these DIRGs. The overlapping DIRGs were selected using the combined LASSO and SVM - RFE methods. ROC curves were constructed to evaluate the predictive ability of these biomarkers, and further validation was performed using additional GEO datasets (GSE14001+GSE73168+GSE146965) and IHC. The composition patterns of LM22 in HGSOC were computed through the CIBERSORT algorithm. Moreover, a correlation analysis was performed between diagnostic immune - related biomarkers and infiltrating immune cells.

It should be noted that within the training cohort, GSE126132 (which contains only HGSOC samples) was combined with GSE27651 (which contains both HGSOC and LGSOC) to perform differential expression analysis. This merged approach allowed robust identification of DEGs with increased HGSOC sample size. For validation, GSE146965 (HGSOC-only) was used to verify the expression trends of candidate biomarkers and the combined validation ROC analysis, although it could not contribute to independent ROC curve analysis due to the absence of LGSOC controls.

### Batch effect removal and cohort validation

We now present the key findings from the batch correction evaluation, referencing **Supplementary Table S1** and **Supplementary Figure S1**. As shown in **Supplementary Figure S3** and quantified in **Supplementary Table S2**, before correction, the median expression levels varied across datasets within each cohort (e.g., training cohort means: GSE27651 7.059, GSE126132 7.026; validation cohort means ranging from 5.37 to 5.43). After applying ComBat separately, all datasets within the training cohort converged to a mean of 7.045 (SD = 0), and those in the validation cohort converged to 5.400 (SD = 0), indicating complete removal of batch effects. Importantly, the biological distinction between the training and validation cohorts was preserved (7.045 vs. 5.400), confirming that the correction did not obscure genuine biological signals and that external validation remains valid.

### Identification of DEGs in HGSOC

**Figure 2:**
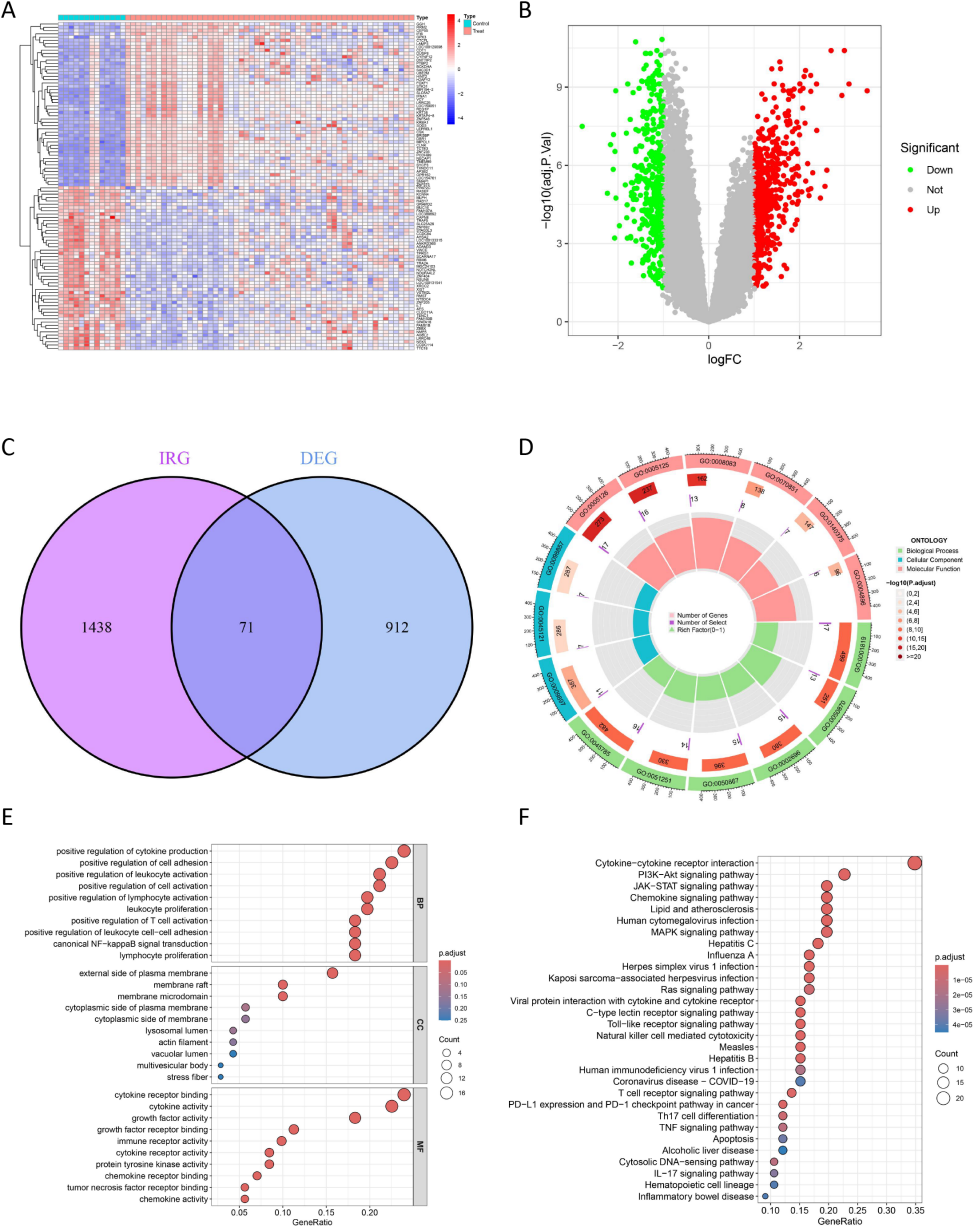
Screening and functional enrichment of DIRGs between HGSOC and LGSOC groups. **(A)** Expression profiling: Heatmap of the top DEGs between HGSOC and LGSOC samples (GEO dataset). **(B)** DEG identification: Volcano plot visualizing 983 DEGs (red, up-regulated; green, down-regulated; black, non-DEG). **(C)** DIRG screening: Venn diagram depicting the overlap of DEGs and immune genes, yielding 71 core DIRGs. **(D, E)** GO enrichment: Circle plot **(D)** and bubble graph **(E)** of Gene Ontology terms for the 71 DIRGs. The complete GO enrichment results, including all terms and their FDR-adjusted q-values. **(F)** Pathway analysis: KEGG pathway annotation results for the identified DIRGs.

In this study, we identified 983 DEGs between 13 LGSOC and 56 HSOG from two datasets (GSE27651 and GSE126132). The selection criteria were an adjusted P - value (adj.P.Val.Filter) < 0.05 and a log fold - change (logFCfilter) = 1 (**Figure 2A**; **Supplementary Table S6**). Specifically, 412 genes were significantly downregulated, while 571 genes were significantly upregulated (**Figure 2B**). To identify DIRGs in HGSOC, we intersected the DEGs with IRGs and found 71 DIRGs (**Figure 2C**; **Supplementary Table S7**).

### GO and KEGG functional enrichment

The results indicated that, in terms of biological processes, the 71 DIRGs were primarily enriched in cytokine-mediated signaling and positive regulation of T cell activation; cellular components were mainly membrane microdomain and external side of plasma membrane; molecular functions included signaling receptor activator activity and immune receptor activity (**Figures 2D, E**; **Supplementary Table S8**). KEGG pathway analysis revealed significant enrichment in cytokine-cytokine receptor interaction, JAK-STAT signaling pathway, Toll-like receptor signaling pathway, and PI3K-Akt signaling pathway (**Figure 2F**; **Supplementary Table S9**). All significantly enriched terms (q < 0.05) are listed in **Supplementary Tables S8**, **S9**, which contain the full statistical details including raw p-values and FDR-adjusted q-values. Collectively, these findings demonstrate a significant association between HGSOC and immunity.

### Constructing the PPI network and selecting hub genes

**Figure 3:**
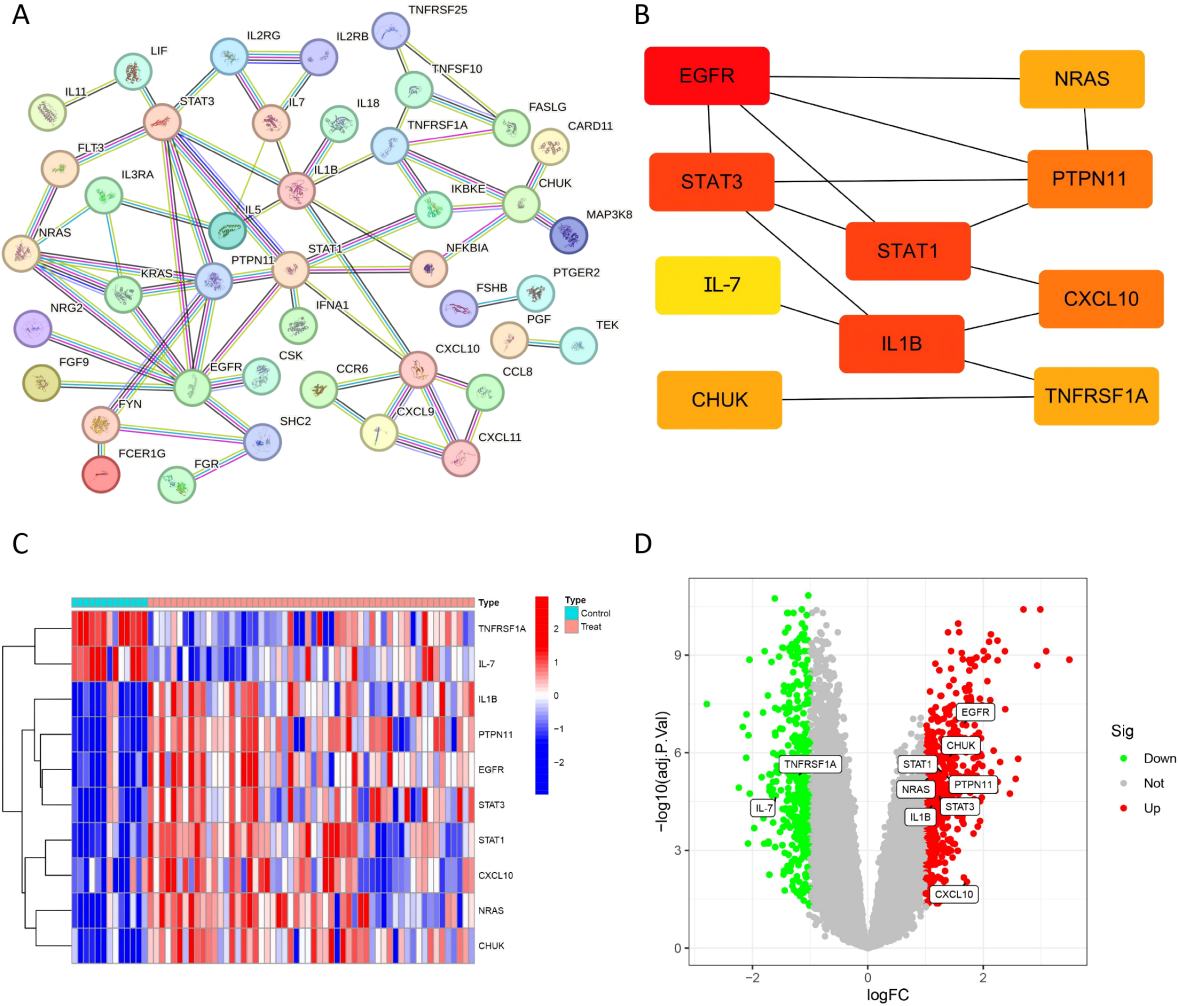
Analysis pipeline for hub gene identification and validation among DIRGs. **(A)** PPI network construction: The interaction network among DIRGs was generated with the STRING tool. **(B)** Hub gene screening: The top five hub genes were identified by applying the Maximal Clique Centrality (MCC) algorithm. **(C, D)** Expression validation: The differential expression patterns of the ten hub genes between HGSOC and LGSOC control samples are displayed as a heatmap **(C)** and a volcano plot **(D)**.

We used the STRING database to explore the PPI among 71 DIRGs associated with HGSOC. Unassociated DIRGs were removed (**Figure 3A**). By clustering the network genes with the CytoHubba plugin, we obtained 10 hub DIRGs based on the Maximal Clique Centrality (MCC) method: *PTPN11*, *STAT1*, *TNFRSF1A*, *NRAS*, *EGFR*, *IL-7*, *STAT3*, *CHUK*, *IL1B*, and *CXCL10* (**Figure 3B**). The expression profiles of all 10 core DIRGs were visualized using a heatmap and a volcano plot. Genes such as *PTPN11*, *STAT1*, *NRAS*, *EGFR*, *STAT3*, *CHUK*, *IL1B*, and *CXCL10* showed high expression levels, while TNFRSF1A and IL-7 genes presented low expression levels (**Figures 3C, D**).

### The associations among the expression intensities of hub genes in HGSOC

**Figure 4:**
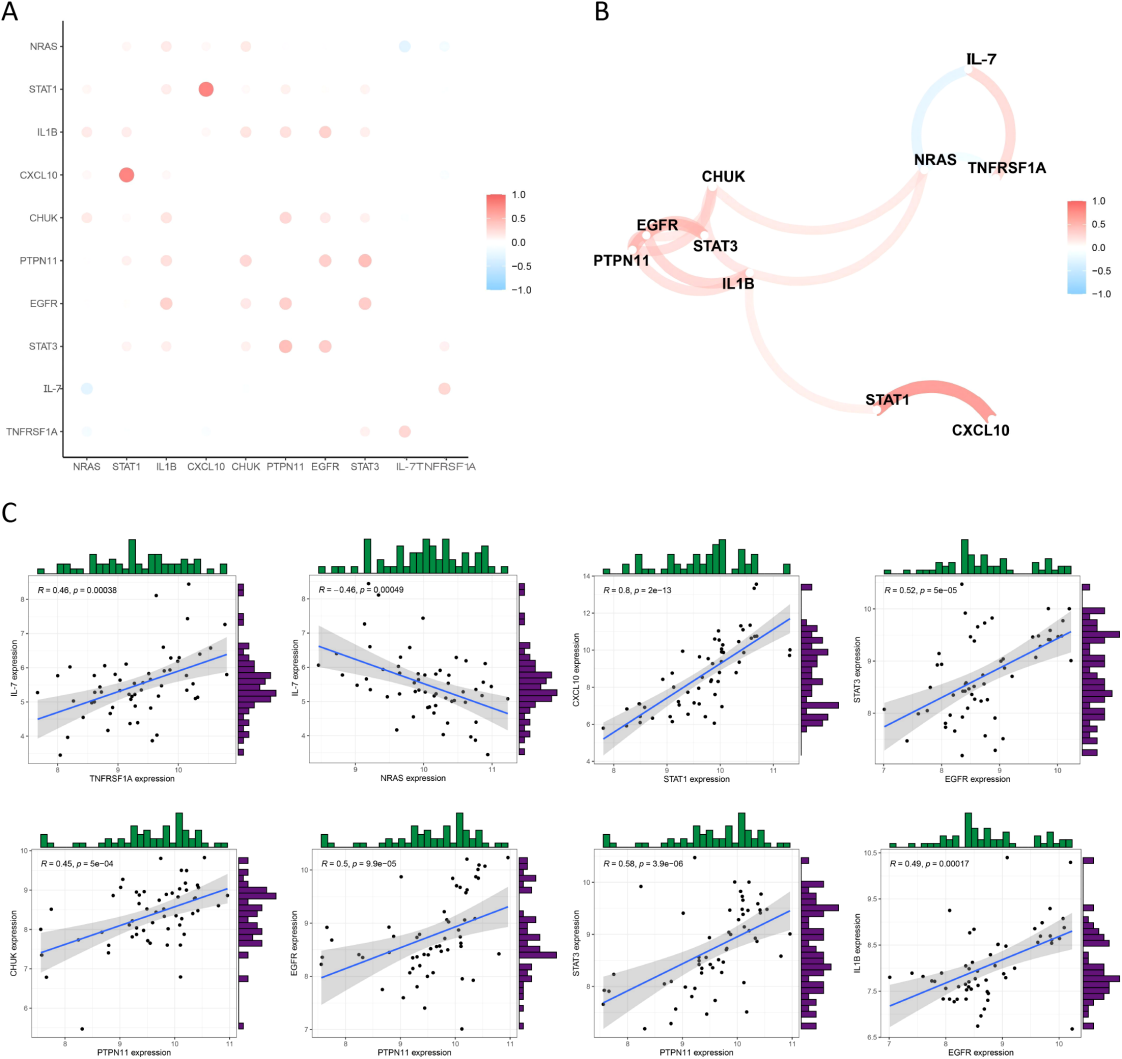
Correlation analysis among DIRGs. **(A)** Network visualization: The co-expression network of DIRGs. **(B)** Correlation assessment: Matrix plot displaying the correlation coefficients between all DIRGs. **(C)** Pairwise validation: Scatter plots confirming the strong linear relationships for specific high-correlation DIRG pairs.

To investigate the potential co-expression relationships among the 10 hub DIRGs, we performed Spearman correlation analysis on their expression levels. A co-expression network map (**Figure 4A**) and a correlation matrix plot (**Figure 4B**) were generated. The strongest positive correlations (r > 0.4, P < 0.001) were observed between pairs such as *PTPN11* and *CHUK*, *EGFR*, or *STAT3*, while *IL-7* showed a negative correlation with *NRAS* and a positive correlation with *TNFRSF1A* (**Figure 4C**).

### Build a diagnostic model for HGSOC

**Figure 5:**
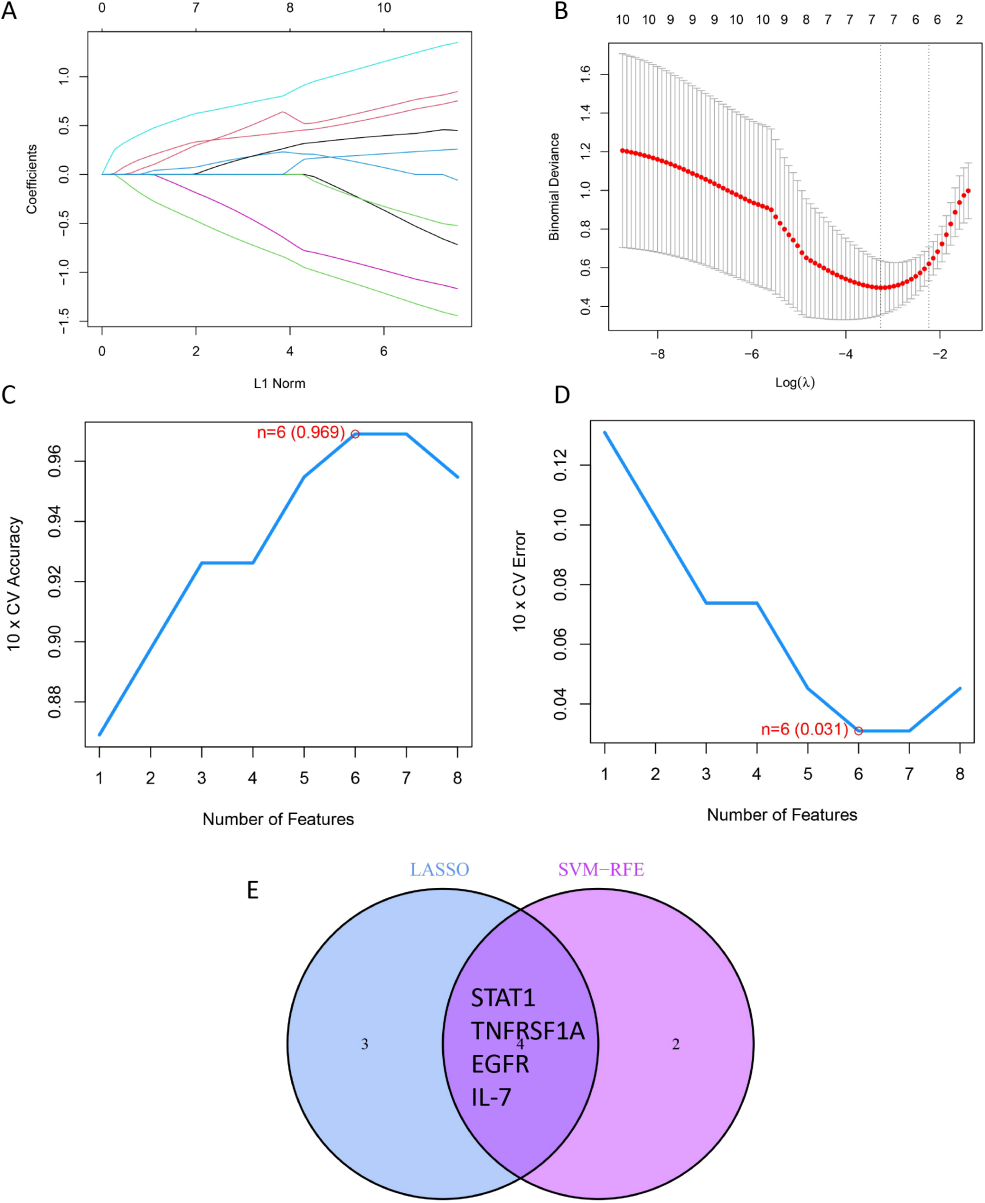
Workflow for constructing a HGSOC prediction model based on DIRGs. **(A)** Feature selection via LASSO: Trajectory of regression coefficients for the 7 DIRGs across a range of lambda values. **(B)** Model tuning: Plot of cross-validated partial likelihood deviance versus log(lambda) to identify the optimal penalization parameter. **(C, D)** Cluster analysis: Determination of the optimal sample cluster number (k=6) using the elbow method **(C)** and silhouette analysis **(D)**. **(E)** Consensus marker identification: Overlap of key features selected by both the LASSO and SVM-RFE algorithms, yielding 4 robust diagnostic markers.

To identify the most robust diagnostic biomarkers from the 10 hub DIRGs, we applied LASSO regression and SVM-RFE algorithms. LASSO regression selected 7 genes (*STAT1*, *TNFRSF1A*, *NRAS*, *EGFR*, *IL-7*, *STAT3*, *CHUK*) (**Figures 5A, B**). SVM-RFE with 10-fold cross-validation identified 6 genes (*EGFR*, *IL-7*, *STAT1*, *TNFRSF1A*, *PTPN11*, *CXCL10*) as the optimal feature set (**Figure 5C, D**). Upon comparing the outcomes of the two algorithms, it was discovered that the final selection included four overlapping candidate genes—*STAT1, TNFRSF1A, EGFR*, and *IL-7*—as shown in **Figure 5E**.

### Additional examination of the four essential DIRGs

**Figure 6:**
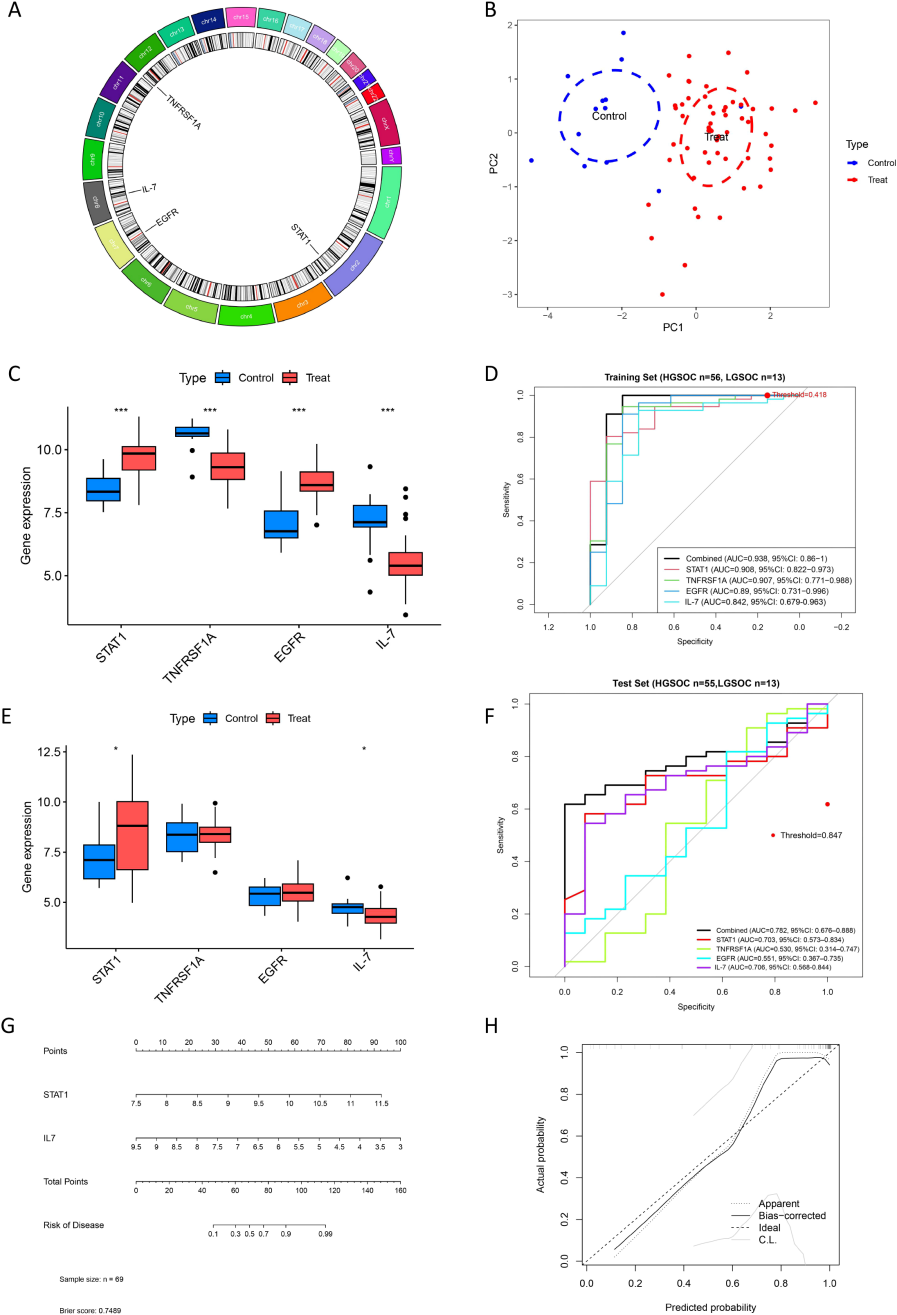
In-depth profiling of four pivotal DIRGs. **(A)** Genomic annotation: Chromosomal locations of the four key DIRGs. **(B)** Sample stratification: PCA plot showing sample clustering driven by the four-gene expression profile. **(C)** The relative expression levels of the four genes between LGSOC and HGSOC from the training group. **(D)** Predictive performance: ROC curve analysis quantifying the diagnostic accuracy of each gene from the training group. **(E)** The relative expression levels of the four genes between LGSOC and HGSOC from the test group. **(F)** ROC curves for *STAT1* and *IL-7* in the combined validation set (GSE14001 + GSE73168 + GSE146965, total n=55 HGSOC, n=13 LGSOC). AUC values: *STAT1* = 0.703, *IL-7* = 0.706 (*P < 0.05). Note that GSE146965 contains only HGSOC samples; separate ROC analyses for datasets containing both subtypes are provided in **Supplementary Figures S8**, **S9**. **(G)** Diagnostic model construction: A two-gene nomogram integrating *STAT1* and *IL-7* for individualized risk prediction. The final model was refined from *STAT1*-*IL-7* gene panel after external validation. **(H)** Calibration curve of a model composed of *STAT1* and *IL-7*.

Four candidate DIRGs (*STAT1*, *TNFRSF1A*, *EGFR*, and *IL-7*), which were identified as common results by both the LASSO regression model and the mSVM - RFE model, were selected for further study. **Figure 6A** shows the chromosomal locations of *STAT1*, *TNFRSF1A*, *EGFR*, and *IL-7*. The results of the principal component analysis suggest that these four candidate genes are highly efficient in distinguishing between HGSOC and LGSOC. This indicates that they may play a crucial role in the diagnosis of HGSOC, as depicted in **Figure 6B**. Moreover, the predictive efficacy of these three genes was evaluated. Compared with LGSOC, *STAT1* and *EGFR* showed up-regulated expression in HGSOC, while *TNFRSF1A* and *IL-7* showed down-regulated expression (**Figure 6C**). To evaluate diagnostic performance, we calculated the AUC for each gene and obtained 95% confidence intervals via bootstrap resampling (1,000 replicates). *STAT1* achieved an AUC of 0.908 (95% CI: 0.822–0.973), *TNFRSF1A* 0.907 (95% CI: 0.771–0.988), *EGFR* 0.890 (95% CI: 0.731–0.996), and *IL-7* 0.842 (95% CI: 0.679–0.963) (**Figure 6D**; **Table 2**). We performed DeLong tests to compare the AUC of the combined model with each individual gene in the training set. The results (**Table 3**) show that the combined model significantly outperformed *IL-7* (p = 0.020), but not *STAT1, TNFRSF1A*, or *EGFR* (p > 0.05). Nevertheless, the combined model yielded the highest numerical AUC, suggesting potential complementary diagnostic value. Using Youden’s index, we determined the optimal probability cutoff (0.418) for the combined model in the training set. At this threshold, the model achieved a sensitivity of 1.000, specificity of 0.846, PPV of 0.966, NPV of 1.000, and accuracy of 0.971. The corresponding confusion matrix (TP = 56, FP = 2, FN = 0, TN = 11) is provided in **Table 4**. The diagnostic performance of *STAT1* and *IL-7* was further evaluated in a combined validation cohort comprising GSE14001 (10 HGSOC + 10 LGSOC), GSE73168 (5 HGSOC + 3 LGSOC), and GSE146965 (40 HGSOC only), totaling 55 HGSOC and 13 LGSOC samples. As depicted in **Figure 6E**, *STAT1* showed up-regulated expression in HGSOC, while *IL-7* showed down-regulated expression. In this merged set, *STAT1* achieved an AUC of 0.703 (95% CI) and *IL-7* achieved an AUC of 0.706 (95% CI) (**Figure 6F**). It should be noted that GSE146965 contains only HGSOC samples; therefore, this merged analysis primarily reflects the capacity of these genes to identify HGSOC within a pooled dataset. To provide more granular validation, we also evaluated diagnostic performance separately in the two datasets containing both subtypes (GSE14001 and GSE73168), with results presented in Result Section “Performance of the combined model in external validation cohorts”.

**Table 2:** Training set AUCs with 95% confidence intervals (bootstrap).

Gene	AUC	95% CI
*STAT1*	0.908	0.822 – 0.973
*TNFRSF1A*	0.907	0.771 – 0.988
*EGFR*	0.890	0.731 – 0.996
*IL-7*	0.842	0.679 – 0.963
Combined	0.938	0.860 – 1.000

**Table 3:** DeLong test comparing combined model vs. individual genes (training set).

Comparison	D value	P value
*STAT1* vs Combined	-0.792	0.429
*TNFRSF1A* vs Combined	-0.493	0.622
*EGFR* vs Combined	-1.396	0.163
*IL-7* vs Combined	-2.322	0.020

**Table 4:** Training set confusion matrix and performance metrics for the combined model at optimal threshold (0.418).

Group	Predicted HGSOC	Predicted LGSOC
Actual HGSOC	56 (TP)	0 (FN)
Actual LGSOC	2 (FP)	11 (TN)

Sensitivity = 1.000, Specificity = 0.846, PPV = 0.966, NPV = 1.000, Accuracy = 0.971.

To further investigate potential overfitting of the four-gene model, we performed learning curve analysis on the training cohort. The learning curves (**Supplementary Figure S5**) showed that as the training sample size increased from 30% to 100%, the cross-validation AUC improved from 0.899 to 0.902, while the training AUC decreased slightly from 0.987 to 0.938 (**Supplementary Table S10**). At the maximum sample size (n=69), the gap between training AUC (0.938) and cross-validation AUC (0.902) was approximately 0.036, indicating minimal overfitting. The dramatic performance drop in GSE73168 (AUC 0.500) is therefore more likely attributable to a combination of factors: (1) the small sample size of GSE73168 (n=8), which makes AUC estimates unstable; (2) instability of inter-gene interaction patterns across cohorts; and (3) potential biological heterogeneity between study populations. Notably, individual genes such as *TNFRSF1A* (AUC 0.881) and *IL-7* (AUC 0.857) retained predictive power in GSE73168, indicating that the information content of the genes was preserved, but the multi-gene combination weights did not generalize. These observations support our decision to refine the diagnostic model to a two-gene panel (*STAT1* and *IL-7*), which demonstrated superior stability and generalizability.

### Refinement to a two-gene diagnostic model with comprehensive calibration assessment

While LASSO regression and SVM-RFE initially identified four overlapping candidate genes (*STAT1, TNFRSF1A, EGFR*, and *IL-7)*, subsequent external validation revealed significant heterogeneity in the diagnostic performance of the multi-gene combination. In the GSE73168 validation cohort, the four-gene combined model exhibited an AUC of only 0.500, with sensitivity 0.786 but specificity 0.000, indicating complete loss of discriminative capacity. Notably, single genes such as *TNFRSF1A* (AUC 0.881) and *IL-7* (AUC 0.857) retained moderate predictive power individually in this cohort, but their combination with other genes failed, suggesting that interaction patterns learned from the training set did not generalize. These findings highlighted the instability of the four-gene panel across independent datasets.

Given these limitations, we systematically evaluated the robustness of each candidate gene across all validation cohorts. *STAT1* and *IL-7* demonstrated the most consistent differential expression patterns: *STAT1* showed significantly higher expression in HGSOC across all datasets, while *IL-7* showed significantly lower expression. In contrast, *TNFRSF1A* and *EGFR* exhibited inconsistent differential expression in certain validation cohorts. Therefore, we constructed a refined two-gene logistic regression model incorporating only *STAT1* and *IL-7.*

Comprehensive calibration assessment was performed as requested. The two-gene model achieved a Brier score of 0.0214, indicating excellent probabilistic predictive accuracy, and an ECE of 0.0707, suggesting minor calibration error (**Supplementary Table S11**). DCA demonstrated positive net benefit across the clinically relevant threshold probability range (0.01–0.99), with net benefit ranging from 0.761 to 0.797 between threshold probabilities of 0.1–0.6 (**Supplementary Figure S6**). The clinically useful threshold probability interval—defined as the range where the model’s net benefit exceeded both “treat all” and “treat none” strategies—spanned 0.01 to 0.99. *Post-hoc* calibration methods (Platt scaling: A = 0.8115, B = 0.3136; and Isotonic regression) did not improve model performance, confirming that the original logistic regression was optimally calibrated.

The nomogram constructed from this two-gene model (**Figure 6G**) provides individualized risk prediction. Notably, *post-hoc* calibration methods (Platt scaling: A = 0.8115, B = 0.3136; and Isotonic regression) did not improve model performance (Brier score 0.0319 and 0.0591, respectively) (**Supplementary Figure S7**), confirming that the original logistic regression was optimally calibrated. The calibration curve demonstrates good agreement between predicted and observed probabilities for the two-gene nomogram, supporting the model’s reliability for clinical risk stratification (**Figure 6H**). This model reduction from four to two genes represents a parsimonious solution that prioritizes robustness and generalizability over complexity, ensuring reliable performance across diverse clinical contexts.

Bootstrap stability analysis (n=1,000) demonstrated exceptional robustness of the two-gene model, with *STAT1* and *IL-7* showing 100% and 100% overall selection frequency, respectively. Independent GSE analysis of GSE27651 confirmed consistent selection of both genes with expected differential expression directions (*STAT1:* logFC=+1.625; *IL-7:* logFC=-2.212) (**Supplementary Figure S8**).

### Performance of the combined model in external validation cohorts

We evaluated the four-gene combined model separately in three independent GEO datasets (GSE14001, GSE73168, GSE146965). GSE146965 contained only HGSOC samples (n=40) and thus could not be used for diagnostic performance assessment, but it confirmed the differential expression of *STAT1* and *IL-7* (see section”Expression validation of *STAT1* and *IL-7* in tissue samples”). The results for GSE14001 and GSE73168 are summarized in **Supplementary Table S12**, and the corresponding ROC curves are provided in **Supplementary Figure S9**; **Supplementary Figure S10**. In GSE14001, the combined model achieved an AUC of 1.000, with sensitivity 1.000, specificity 0.800, PPV 0.333, NPV 1.000, and accuracy 0.818 at the training-derived threshold (0.418). The low PPV likely reflects the small number of LGSOC samples in this cohort (n=10 LGSOC, of which 2 were misclassified as HGSOC at the threshold). In GSE73168, the combined model’s AUC dropped to 0.500, with sensitivity 0.786, specificity 0.000, PPV 0.786, NPV 0.000, and accuracy 0.647. Notably, single genes such as *TNFRSF1A* (AUC 0.881) and IL-7 (AUC 0.857) retained moderate predictive power, but their combination failed, suggesting that the interaction patterns learned from the training set may not generalize to this cohort. These results highlight the heterogeneity across ovarian cancer datasets and underscore the need for cautious interpretation of multi-gene models. The instability of the combined model in GSE73168 suggests potential overfitting or biological differences (e.g., tumor purity, platform effects) that warrant further investigation.

### Expression validation of *STAT1* and *IL-7* in tissue samples

**Figure 7:**
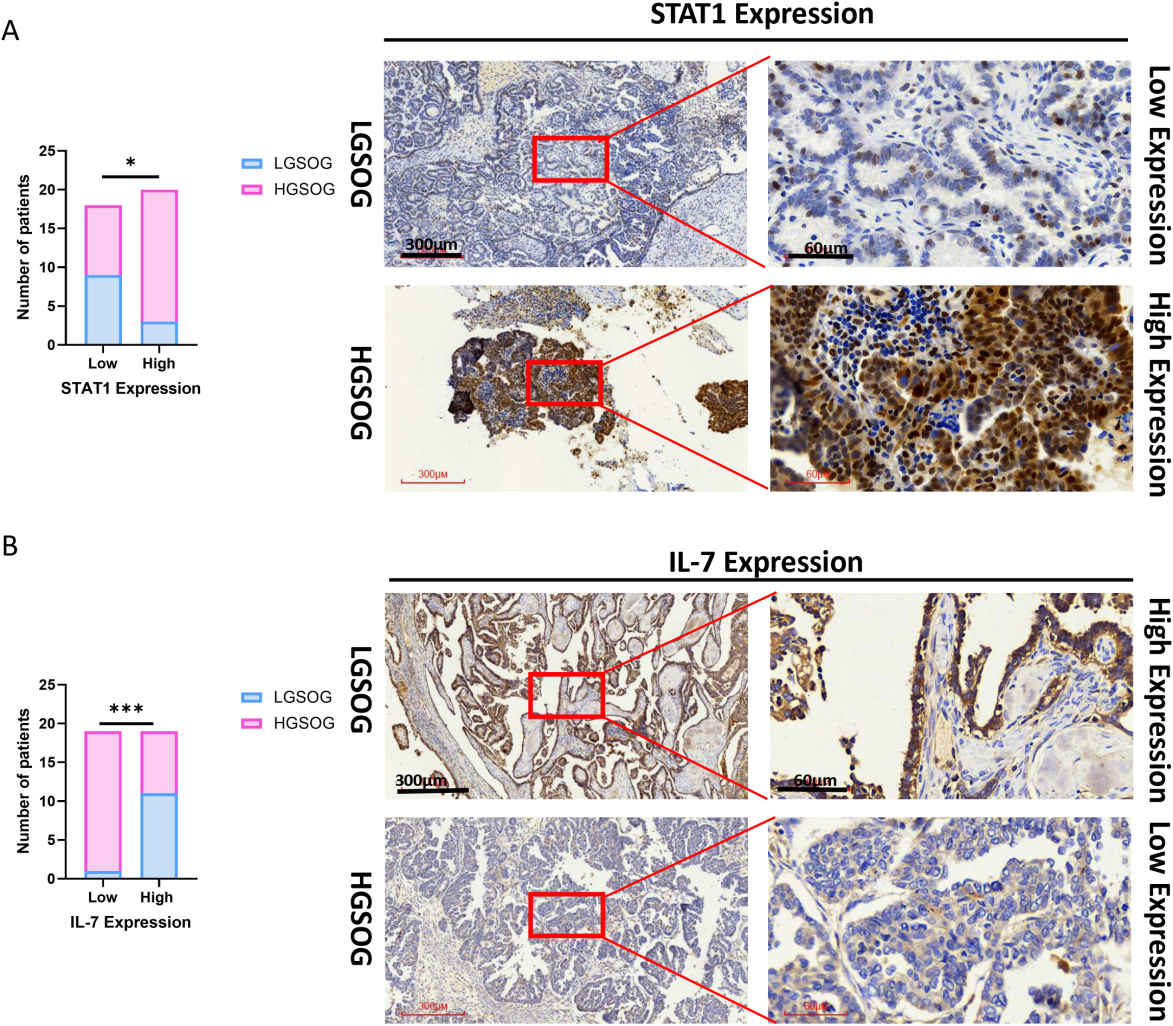
IHC validation of candidate diagnostic biomarkers. **(A)** Validation of *STAT1* expression: IHC analysis reveals significantly higher expression of *STAT1* in HGSOC tissues (n=26) compared to LGSOC controls (n=12). **(B)** Validation of *IL-7* expression: Significantly downregulated *IL-7* expression in HGSOC tissues compared to LGSOC controls. Representative IHC staining images (×40 and ×200) are displayed for each gene. *P < 0.05; ***P < 0.001.

To further validate the expression patterns of the two key diagnostic biomarkers, we examined *STAT1* and *IL-7* expression in our own tissue samples by immunohistochemistry (IHC). IHC analysis of 26 HGSOC and 12 LGSOC tissue specimens confirmed these findings. Using the ROC−derived cutoffs (*STAT1* ≥4, *IL−7* ≥5; **Supplementary Figure S4**; **Supplementary Table S13**), *STAT1* high expression was observed in 17/26 (65.4%) HGSOC versus 3/12 (25.0%) LGSOC (Chi−square test, P = 0.0205; **Figure 7A**), while *IL−7* high expression was observed in 5/26 (19.2%) HGSOC versus 9/12 (75.0%) LGSOC ((Chi−square test, P = 0.0005; **Figure 7B**), consistent with the transcriptomic findings. Representative IHC staining images are shown in **Figure 7**. These results provide independent experimental validation that *STAT1* and *IL-7* are differentially expressed at the protein level and support their potential as diagnostic biomarkers for distinguishing HGSOC from LGSOC.

### Distribution of immune cells in HGSOC and diagnostic genes

**Figure 8:**
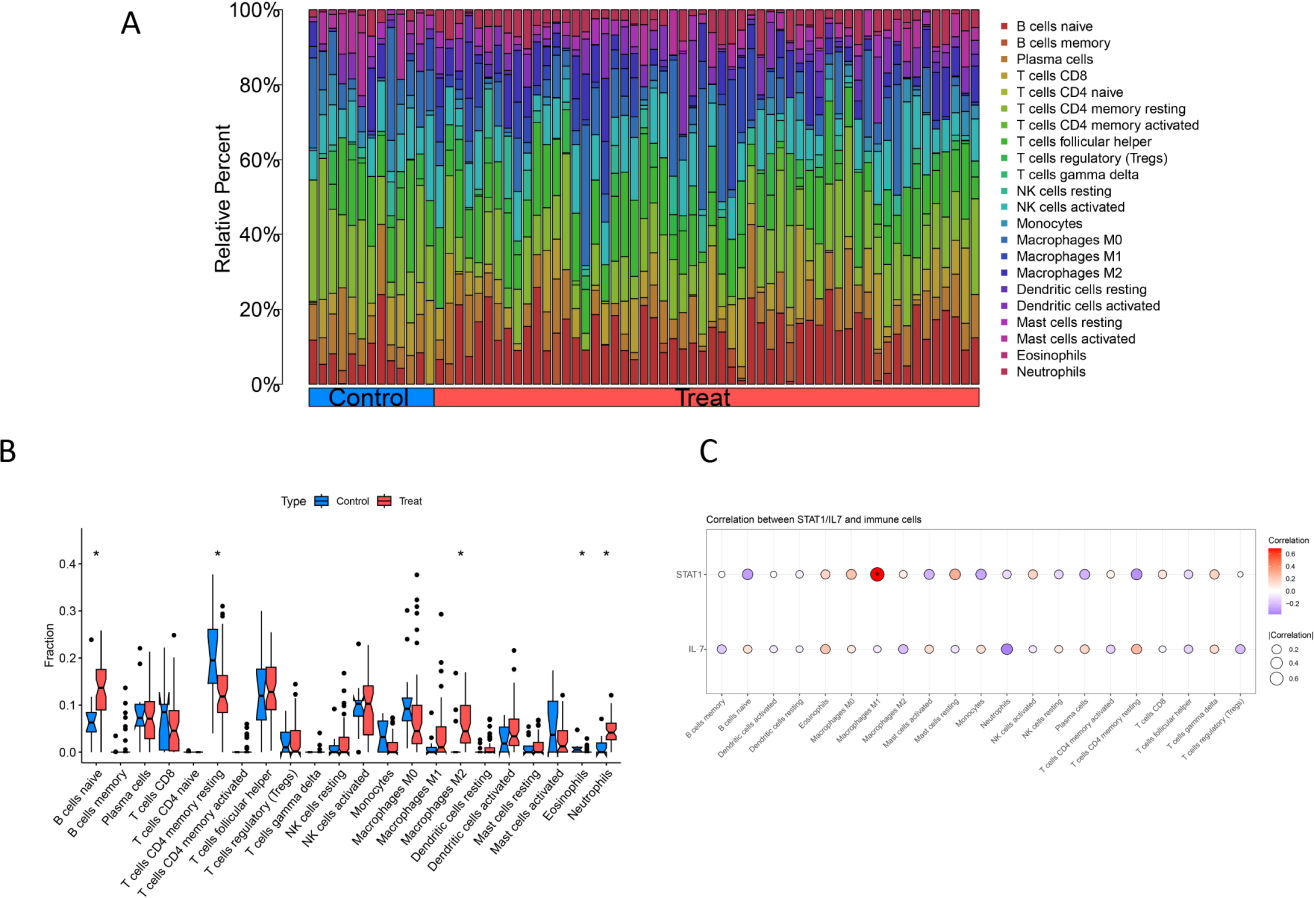
Association of the immune microenvironment with *STAT1* and *IL-7* expression. **(A)** Comparative Analysis: Stacked bar plot illustrating the composition of 22 immune cell types in HGSOC versus LGSOC. **(B)** Boxplots showing the relative abundances of 22 immune cell types between HGSOC (n=56) and LGSOC (n=13) samples. * indicates FDR-adjusted q < 0.05 (Mann–Whitney U test with Benjamini–Hochberg correction). Monocytes showed a lower fraction in HGSOC (raw P = 0.026), but this did not remain significant after multiple testing correction (FDR q = 0.095). **(C)** Bubble plot of Spearman correlations between *STAT1/IL-7* expression and 22 immune cell types in HGSOC samples. The size of each bubble is proportional to the absolute value of the correlation coefficient (|ρ|), and the color represents the direction and strength of the correlation (blue: negative; red: positive). Asterisks (*) indicate correlations that remained statistically significant after Benjamini–Hochberg FDR correction (q < 0.05). Only the *STAT1*–M1 macrophage correlation passed this threshold.

Finally, we evaluated the expression levels of the *STAT1* and *IL-7* genes and explored their relationship with the extent of immune cell infiltration. By applying the CIBERSORT algorithm, we quantified the relative abundances of 22 distinct types of immune cells in both HGSOC and LGSOC samples (**Figure 8A**; **Supplementary Table S14**). Compared with LGSOC, the HGSOC group exhibited significantly higher fractions of naïve B cells (FDR q = 0.0107), M2 macrophages (FDR q = 0.0107), and neutrophils (FDR q = 0.00336), while resting memory CD4+ T cells (FDR q = 0.0107) and eosinophils (FDR q = 0.0107) were significantly lower after FDR correction. Monocytes also showed a lower fraction in HGSOC (raw P = 0.0259), but this difference did not remain statistically significant after multiple testing correction (FDR q = 0.0949) (**Figure 8B**). Detailed statistics for all 22 immune cell types are provided in **Supplementary Table S15**. To assess the relationship between the two diagnostic biomarkers and the immune microenvironment, we analyzed the correlation of *STAT1* and *IL−7* expression with the abundances of 22 immune cell subsets estimated by CIBERSORT, and cross−validated the key findings using the xCell algorithm. As shown in **Figure 8C** (CIBERSORT results), *STAT1* exhibited a strong positive correlation with M1 macrophages (ρ=0.688, FDR q=2.45×10^−7^). This association was confirmed by xCell (ρ=0.457, FDR q=0.0102), indicating a robust link between *STAT1* expression and M1 macrophage infiltration in HGSOC. No other immune cell types remained significantly correlated with *STAT1* after FDR correction in either algorithm.

*IL−7* showed a negative correlation with neutrophils in CIBERSORT (ρ=−0.372, raw P=0.0048), but this association did not survive FDR correction (q=0.083) and was not replicated by xCell (ρ=0.156, P = 0.250, FDR q=0.588). Thus, the *IL−7*–neutrophil relationship was not consistent across algorithms and did not reach statistical significance after multiple testing correction.

Full correlation results for all 22 cell types and both algorithms are provided in **Supplementary Table S16**, and visualized in **Supplementary Figure S11**. These findings demonstrate that *STAT1* is consistently associated with M1 macrophages, whereas *IL−7* lacks a robust immune correlate in the HGSOC microenvironment.

## Discussion

This multi-cohort study identifies *STAT1* and *IL-7* as robust biomarkers distinguishing HGSOC from LGSOC, validated at both the transcriptomic and protein levels. Machine learning on training cohorts initially selected four genes, but external validation refined this to a two-gene panel (*STAT1* and *IL-7*) with consistent differential expression across independent datasets and IHC confirmation. Immune deconvolution revealed that HGSOC exhibits a distinct microenvironment characterized by higher fractions of naïve B cells, M2 macrophages, and neutrophils, and lower fractions of resting memory CD4+ T cells and eosinophils compared to LGSOC. Notably, *STAT1* expression demonstrated a strong, reproducible correlation with M1 macrophages, a finding cross-validated by two independent algorithms (CIBERSORT and xCell). These findings position *STAT1* and *IL-7* not only as potential diagnostic adjuncts in histologically ambiguous cases but also as reflectors of the profoundly different immune landscapes that define these two ovarian cancer subtypes.

Consistent with our enrichment analysis, which highlighted cytokine-cytokine receptor interaction and JAK-STAT pathways, the identified DIRGs are central to immune signaling. The convergence of our bioinformatic screening and machine learning on *STAT1* and *IL-7* is particularly compelling. *IL-7*, a stroma-derived cytokine, is crucial for T-cell homeostasis (**Supplementary Figure S12**) (17). Its significant downregulation in HGSOC may impair T-cell survival and function, contributing to the immunosuppressive microenvironment characteristic of this aggressive subtype. This aligns with recent reports that targeting the *IL-7* receptor can reverse M2-like macrophage polarization and suppress tumor growth in ovarian cancer (18). Conversely, the higher expression of *IL-7* in LGSOC may reflect its “colder” immune phenotype, where stromal production of *IL-7* represents a compensatory, albeit ineffective, mechanism to sustain a limited T-cell pool (11, 15).

The role of STAT1 in HGSOC is more nuanced. As a canonical tumor suppressor and effector of IFN-γ signaling, its strong upregulation in HGSOC seems paradoxical (19). However, our single-cell query using the TISCH database (https://tisch.compbio.cn/search-gene/) clarifies that *STAT1* expression is predominantly confined to immune cells, particularly macrophages and T cells, rather than the malignant epithelium (**Supplementary Figure S12**). Therefore, the high *STAT1* expression in HGSOC bulk tumors reflects the enrichment of these immune populations. Its robust correlation with M1 macrophages, confirmed by both CIBERSORT and xCell, reinforces its role as a marker of an inflamed, or “hot,” tumor microenvironment. Yet, in the context of near-universal TP53 mutations and genomic instability that drive chronic inflammation in HGSOC, this sustained *STAT1* activation may be maladaptive (12, 20). It could contribute to T-cell exhaustion and immune evasion, meaning the abundant M1 macrophages may be functionally impaired (21, 22). Thus, the STAT1-M1 correlation in HGSOC may signify a tumor-hijacked inflammatory response rather than effective anti-tumor immunity, a hypothesis supported by the well-documented STAT1/STAT3 imbalance that favors tumor progression in this cancer type (19, 23, 24).

To contextualize the diagnostic potential of *STAT1* and *IL-7*, it is essential to compare them with established immunohistochemical markers. In clinical practice, HGSOC and LGSOC are distinguished by histopathology supported by p53, WT1, and Ki-67: HGSOC typically shows aberrant p53 expression and a high proliferative index (12, 25). While p53 aberrancy remains a hallmark, its interpretation can be inconclusive in 5-10% of cases. In such equivocal scenarios—or in small biopsy specimens where tissue architecture is limited—*STAT1* and *IL-7* may offer ancillary value. The upregulation of STAT1 and downregulation of IL-7 provide a distinct molecular signature reflecting the underlying immune pathway activation that differentiates HGSOC from LGSOC (14). However, these findings are exploratory; the diagnostic gain of incorporating these markers into routine pathology requires prospective validation in cohorts enriched for challenging cases, including borderline tumors.

The strong correlation between *STAT1* and M1 macrophages, alongside the downregulation of *IL-7* in an M2-dominant environment, has potential implications for immunotherapy. The IL-7/IL-7R axis is emerging as a promising therapeutic target. Inhibiting IL-7R has been shown to suppress tumor growth and reverse M2 polarization, suggesting that the low *IL-7* expression we observe in HGSOC might be part of a broader immunosuppressive program that could be therapeutically reversed (18). For *STAT1*, its role as a marker of an inflamed microenvironment suggests that *STAT1*-high HGSOC might be more amenable to immune checkpoint blockade. However, the potential for T-cell exhaustion in this context also implies that combining checkpoint inhibitors with agents that reinvigorate exhausted T-cells could be particularly effective (10, 21).

Several limitations of this study should be acknowledged. First, our reliance on publicly available datasets, with modest sample sizes, particularly for IHC validation, means our findings require confirmation in larger, prospective cohorts. Second, the diagnostic performance of the four-gene combined model was inconsistent across external cohorts, highlighting significant inter-cohort heterogeneity and the risk of overfitting in multi-gene models. This led us to refine our focus to the more robust two-gene panel. Third, while we used two deconvolution algorithms, incorporating additional methods could provide further granularity. Finally, as a retrospective bioinformatics study, the clinical utility of *STAT1* and *IL-7* as ancillary diagnostic tools in truly ambiguous cases remains to be established.

## Conclusion

In summary, this study identifies *STAT1* and IL-7 as consistently differentially expressed genes between HGSOC and LGSOC, with validation at both the transcriptomic and protein levels. Their expression patterns are closely linked to the distinct immune microenvironments of these two subtypes, with *STAT1* robustly correlating with M1 macrophages. While they are not intended to replace established diagnostic markers, they may hold potential as ancillary biomarkers in histologically ambiguous cases. Furthermore, these findings provide a rationale for exploring the IL-7/IL-7R axis and the *STAT1*-driven immune contexture as potential guides for future immunotherapeutic strategies in HGSOC. Prospective studies are now warranted to validate their clinical utility and further elucidate the underlying biological mechanisms.

## Data Availability

The original contributions presented in the study are included in the article/**Supplementary Material**. Further inquiries can be directed to the corresponding author.

## Supplementary material

Supplementary Figure S1 PCA of gene expression profiles before and after batch correction. **(A)** Training set before ComBat correction, points colored by original dataset (batch). **(B)** Training set after ComBat correction. **(C)** Test set before correction. **(D)** Test set after correction. **(E)** Combined training and test sets after separate batch correction, colored by cohort. The R² and P values shown on each panel are derived from PERMANOVA testing the effect of batch **(A–D)** or cohort **(E)**. Note that batch effects are almost completely removed within each cohort (R² ≈ 0, P = 1), while a residual biological difference remains between training and test sets (R² = 0.577, P = 0.001), justifying the need for external validation.

Supplementary Figure S2 RLE plots before and after correction for each cohort.

Supplementary Figure S3 Boxplots of median gene expression per sample before and after ComBat batch correction. **(A)** Training cohort (GSE27651, GSE126132). **(B)** Validation cohort (GSE14001, GSE73168, GSE146965). For each sample, the median expression value across all genes was calculated. Boxes represent the interquartile range (IQR) with the median indicated by the horizontal line; whiskers extend to the most extreme data points within 1.5 × IQR. Individual data points are overlaid as jittered dots. Orange and blue boxes represent samples before and after correction, respectively. After correction, the median expression levels become identical across all datasets within each cohort (all means converge to 7.045 in the training set and 5.400 in the validation set), demonstrating effective removal of batch effects while preserving biological differences between cohorts. Summary statistics are provided in **Supplementary Table S2**.

Supplementary Figure S4 ROC curves for determining IHC cutoffs. ROC curves for *STAT1* (A) and *IL-7* (B) based on IHC scores from 26 HGSOC and 12 LGSOC samples. The optimal cutoffs (Youden index) were 3.5 for *STAT1* (sensitivity 65.4%, specificity 75.0%, AUC = 0.716) and 5.0 for *IL-7* (sensitivity 69.2%, specificity 91.7%, AUC = 0.817). The nearest integer values (*STAT1* ≥4, *IL−7* ≥5) were adopted as the final cutoffs for high expression.

Supplementary Figure S5 Learning curves for the four-gene logistic regression model, showing training and cross-validation AUC as a function of training sample size (based on 10 repeats of 5-fold CV).

Supplementary Figure S6 Decision curve analysis for the two-gene nomogram (*STAT1* + *IL-7*). Net benefit across threshold probabilities (0–1) for the two-gene model (blue), “treat all” (green dashed), and “treat none” (gray dashed). The model shows positive net benefit across 0.01–0.99, with stable performance (0.76–0.80) at 0.1–0.6.

Supplementary Figure S7 Calibration curve comparison demonstrating that the original two-gene logistic regression model (blue; Brier score 0.0214) outperforms *post-hoc* calibration methods including Platt scaling (green; Brier score 0.0319) and isotonic regression (red; Brier score 0.0591), with bubble size indicating sample distribution across probability bins.

Supplementary Figure S8 Bootstrap stability analysis (n=1,000) demonstrating 100% overall selection frequency for both *STAT1* and *IL-7* across LASSO regression and SVM-RFE algorithms, confirming the robustness of the two-gene diagnostic signature.

Supplementary Figure S9 ROC curves for the four-gene combined model and individual genes in the GSE14001 validation cohort. The plot shows ROC curves for *STAT1*, *TNFRSF1A*, *EGFR*, *IL-7*, and their logistic regression combination. The area under the curve (AUC) values are: Combined = 1.000, *STAT1* = 1.000, *TNFRSF1A* = 0.800, *EGFR* = 0.700, *IL-7* = 0.900.

Supplementary Figure S10 ROC curves for the four-gene combined model and individual genes in the GSE73168 validation cohort. ROC curves for *STAT1*, *TNFRSF1A*, *EGFR, IL-7*, and the combined model in the GSE73168 dataset. AUC values: Combined = 0.500, *STAT1* = 0.595, *TNFRSF1A* = 0.881, *EGFR* = 0.690, *IL-7* = 0.857.

Supplementary Figure S11 Correlation of *STAT1* and *IL-7* expression with immune cell fractions estimated by CIBERSORT and xCell in HGSOC. **(A)** Bubble plot showing Spearman correlation between *STAT1* expression and the abundance of 22 immune cell types, as estimated by CIBERSORT (triangle) and xCell (circle). The size of each bubble is proportional to the absolute value of the correlation coefficient (|ρ|), and the color represents the direction and strength of correlation (red: positive; blue: negative). Asterisks denote statistical significance: ** FDR q<0.05; * raw P<0.05 but FDR q≥0.05. **(B)** Corresponding bubble plot for IL-7. Only HGSOC samples (n=56) were included in the analysis.

Supplementary Figure S12 *STAT1* and *IL-7* expression at single-cell resolution using publicly available datasets via the TISCH database.

Supplementary Table S1 PERMANOVA results quantifying batch effects before and after ComBat correction.

Supplementary Table S2 Summary statistics of sample median expression before and after batch correction. For each cohort (Training, Validation), dataset (GSE accession), and correction stage (Before correction, after correction), the mean, standard deviation (SD), minimum, maximum, and number of samples (n) of per−sample median expression values are presented. After correction, all datasets within the same cohort exhibit identical means and zero SD, confirming successful batch effect removal.

Supplementary Table S3 The immune-related genes (IRGs) from the Import database.

Supplementary Table S4 ROC analysis results for IHC cutoffs.

Supplementary Table S5 Key R packages, versions, and parameters used in this study.

Supplementary Table S6 983 DEGs between HGSOC and LGSOC controls within the two datasets (GSE27651+ GSE126132).

Supplementary Table S7 71 DIRGs were found by intersecting the DEGs and IRGs.

Supplementary Table S8 Complete results of GO enrichment analysis.

Supplementary Table S9 Complete results of KEGG pathway enrichment analysis.

Supplementary Table S10 Summary of learning curve results at each training fraction (mean AUC ± SD, n=10 repeats).

Supplementary Table S11 Comprehensive calibration and clinical utility metrics for the two-gene diagnostic nomogram (*STAT1* + *IL-7*).

Supplementary Table S12 External validation results for each GEO dataset.

Supplementary Table S13 HGSOC vs LGSOC clinical characteristics.

Supplementary Table S14 The relative abundance of the 22 immune cell subtypes in HGSOC and LGSOC specimens.

Supplementary Table S15 immune cell proportion comparisons with FDR.

Supplementary Table S16 correlation results for all 22 cell types and both algorithms.
