# Supplementary material for: STAT1 and IL-7 as potential diagnostic biomarkers for distinguishing high-grade from low-grade serous ovarian cancer: a multi-cohort analysis
Source: Front Immunol. 2026 Apr 14;17:1779912. doi: 10.3389/fimmu.2026.1779912 (PMC13120972; doi:10.3389/fimmu.2026.1779912)
Supplement: Supplementary Figure S1 — PCA of gene expression profiles before and after batch correction. (A) Training set before ComBat correction, points colored by original dataset (batch). (B) Training set after ComBat correction. (C) Test set before correction. (D) Test set after correction. (E) Combined training and test sets after separate batch correction, colored by cohort. The R² and P values shown on each panel are derived from PERMANOVA testing the effect of batch (A–D) or cohort (E). Note that batch effects are almost completely removed within each cohort (R² ≈ 0, P = 1), while a residual biological difference remains between training and test sets (R² = 0.577, P = 0.001), justifying the need for external validation. [file DataSheet1.zip › revised supplementary/Table S12. External validation results for each GEO dataset.docx]

#### ****Table S11. External validation results for each GEO dataset****

| Dataset | Sample size (HGSOC/LGSOC) | Model | AUC | Sensitivity | Specificity | PPV | NPV | Accuracy |
| --- | --- | --- | --- | --- | --- | --- | --- | --- |
| GSE14001 | 10 / 10 | Combined | 1.000 | 1.000 | 0.800 | 0.333 | 1.000 | 0.818 |
| GSE73168 | 5 / 3 | Combined | 0.500 | 0.786 | 0.000 | 0.786 | 0.000 | 0.647 |
| GSE146965* | 40 / 0 | – | – | – | – | – | – | – |

*GSE146965 contained only HGSOC samples and was used only for expression validation (Figure 7A). When all three validation datasets were pooled, the combined model achieved an AUC of 0.716 (data not shown), consistent with the per-dataset results.*
