# Supplementary material for: STAT1 and IL-7 as potential diagnostic biomarkers for distinguishing high-grade from low-grade serous ovarian cancer: a multi-cohort analysis
Source: Front Immunol. 2026 Apr 14;17:1779912. doi: 10.3389/fimmu.2026.1779912 (PMC13120972; doi:10.3389/fimmu.2026.1779912)
Supplement: Supplementary Figure S1 — PCA of gene expression profiles before and after batch correction. (A) Training set before ComBat correction, points colored by original dataset (batch). (B) Training set after ComBat correction. (C) Test set before correction. (D) Test set after correction. (E) Combined training and test sets after separate batch correction, colored by cohort. The R² and P values shown on each panel are derived from PERMANOVA testing the effect of batch (A–D) or cohort (E). Note that batch effects are almost completely removed within each cohort (R² ≈ 0, P = 1), while a residual biological difference remains between training and test sets (R² = 0.577, P = 0.001), justifying the need for external validation. [file DataSheet1.zip › revised supplementary/Table S1PERMANOVA results quantifying batch effects before and after ComBat correction.docx]

| ****Cohort**** | ****Phase**** | ****R²**** | ****P-value**** |
| --- | --- | --- | --- |
| Training set | Before batch correction | 0.546 | 0.001 |
| Training set | After batch correction | 8.05×10⁻⁵ | 1 |
| Test set | Before batch correction | 0.748 | 0.001 |
| Test set | After batch correction | 1.09×10⁻⁴ | 1 |
| Train vs Test | After separate correction | 0.577 | 0.001 |

Table S1. PERMANOVA results quantifying batch effects before and after ComBat correction.
