# Supplementary material for: STAT1 and IL-7 as potential diagnostic biomarkers for distinguishing high-grade from low-grade serous ovarian cancer: a multi-cohort analysis
Source: Front Immunol. 2026 Apr 14;17:1779912. doi: 10.3389/fimmu.2026.1779912 (PMC13120972; doi:10.3389/fimmu.2026.1779912)
Supplement: Supplementary Figure S1 — PCA of gene expression profiles before and after batch correction. (A) Training set before ComBat correction, points colored by original dataset (batch). (B) Training set after ComBat correction. (C) Test set before correction. (D) Test set after correction. (E) Combined training and test sets after separate batch correction, colored by cohort. The R² and P values shown on each panel are derived from PERMANOVA testing the effect of batch (A–D) or cohort (E). Note that batch effects are almost completely removed within each cohort (R² ≈ 0, P = 1), while a residual biological difference remains between training and test sets (R² = 0.577, P = 0.001), justifying the need for external validation. [file DataSheet1.zip › revised supplementary/Table S5. Key R packages, versions, and parameters used in this study.docx]

**Table S5. Key R packages, versions, and parameters used in this study**

*Note: Note: R packages marked with R version 4.1.3 were used for primary analyses including data preprocessing, differential expression analysis, functional enrichment, and immune infiltration analysis. R packages marked with R version 4.3.3 were used for additional validation analyses including batch effect assessment, biomarker evaluation, nomogram validation, bootstrap stability analysis, and IHC scoring.*

| **Analysis step** | **R package / Tool** | **Version¹** | **R Version** | **Key parameters / settings** |
| --- | --- | --- | --- | --- |
| **Data preprocessing and batch effect removal** | limma | 3.50.0 | 4.1.3 | avereps (collapse multiple probes to mean), normalizeBetweenArrays (quantile normalization) |
|  | sva | 3.42.0 | 4.1.3 | ComBat batch correction, applied separately to training and validation cohorts; parametric adjustment, empirical Bayes |
| **Differential expression analysis** | limma | 3.50.0 | 4.1.3 | lmFit, eBayes; criteria: \|log2FC\| >= 1, adjusted P < 0.05 (Benjamini-Hochberg) |
| **Functional enrichment analysis** | clusterProfiler | 4.2.2 | 4.1.3 | enrichGO, enrichKEGG; p-value cutoff (raw) = 0.05, q-value cutoff (FDR) = 0.05 |
|  | org.Hs.eg.db | 3.14.0 | 4.1.3 | Human genome annotation |
|  | enrichplot | 1.14.2 | 4.1.3 | Visualization of enrichment results |
|  | DOSE | 3.20.1 | 4.1.3 | Disease ontology enrichment |
| **Machine learning feature selection** | glmnet | 4.1-7 | 4.1.3 | LASSO logistic regression (family = "binomial"); 10-fold cross-validation; lambda.min selected |
|  | e1071 | 1.7-13 | 4.1.3 | SVM-RFE with linear kernel (kernel = "linear", cost = 1); 10-fold cross-validation |
| **Machine learning and model evaluation** | caret | 6.0-94 | 4.3.3 | Data partitioning (createDataPartition), confusion matrix (confusionMatrix), cross-validation folds (createFolds) |
| **Diagnostic model evaluation** | pROC | 1.18.0 | 4.1.3 | ROC curve, AUC calculation; 95% CI by bootstrap (1000 replicates) or DeLong method; Youden index for optimal threshold |
|  | boot | 1.3-28.1 | 4.3.3 | Bootstrap resampling (R=1000) for calculating 95% confidence intervals of AUC |
| **Probability calibration** | MASS | 7.3-60 | 4.3.3 | Statistical functions for probability calibration analysis (qlogis, plogis) |
| **Nomogram construction and validation** | rms | 6.3-0 | 4.1.3 | Logistic regression model (lrm); nomogram; calibration curves (calibrate with 1000 bootstrap) |
|  | rmda | 1.6 | 4.1.3 | Decision curve analysis (dca); net benefit calculation; bootstraps = 50 |
| **Immune infiltration analysis** | xCell | 1.1.0 | 4.1.3 | Default parameters (uses AUC for enrichment scores); immune cell types overlapping with CIBERSORT retained |
| **Data manipulation** | dplyr | 1.1.4 | 4.3.3 | Data manipulation (filter, select, mutate, %>% pipe operator, etc.) |
|  | reshape2 | 1.4.4 | 4.3.3 | Data reshaping (melt, dcast) for ggplot2 long-format data preparation |
|  | stringr | 1.5.1 | 4.3.3 | String processing (str_split, str_detect, etc.) for sample name parsing |
| **Data I/O** | openxlsx | 4.2.5.2 | 4.3.3 | Read/write Excel files (.xlsx format) for IHC data output |
|  | readxl | 1.4.3 | 4.3.3 | Read Excel files (.xls/.xlsx format) for IHC data input |
| **Visualization** | ggplot2 | 3.3.6 | 4.1.3 | Boxplots, bar plots, bubble plots, etc. |
|  | corrplot | 0.92 | 4.1.3 | Correlation matrix visualization |
|  | vioplot | 0.3.7 | 4.1.3 | Violin plots for immune cell comparisons |
|  | ggpubr | 0.6.0 | 4.3.3 | Statistical graphics and publication-ready plots (stat_compare_means, etc.) |
|  | patchwork | 1.1.3 | 4.3.3 | Multi-plot layout composition (e.g., RLE plots: (p1 \| p2) / (p3 \| p4)) |
| **PERMANOVA / batch effect assessment** | vegan | 2.6-2 | 4.1.3 | adonis2 for permutational multivariate analysis of variance; 999 permutations |

**Legend:**R 4.1.3 = Main analysis pipeline; R 4.3.3 = Uploaded R scripts. All Bioconductor packages for R 4.1.3 are from Bioconductor 3.14. CRAN package versions follow standard R version numbering (e.g., 4.1-7).

¹Version numbering: Bioconductor packages use x.y.z format (e.g., 3.50.0); CRAN packages may use x.y-z format (e.g., 4.1-7) or x.y.z format. MASS and boot are part of the R recommended packages and their versions correspond to specific R releases.
