# Supplementary figures and images for: STAT1 and IL-7 as potential diagnostic biomarkers for distinguishing high-grade from low-grade serous ovarian cancer: a multi-cohort analysis
Source: Front Immunol. 2026 Apr 14;17:1779912. doi: 10.3389/fimmu.2026.1779912 (PMC13120972; doi:10.3389/fimmu.2026.1779912)

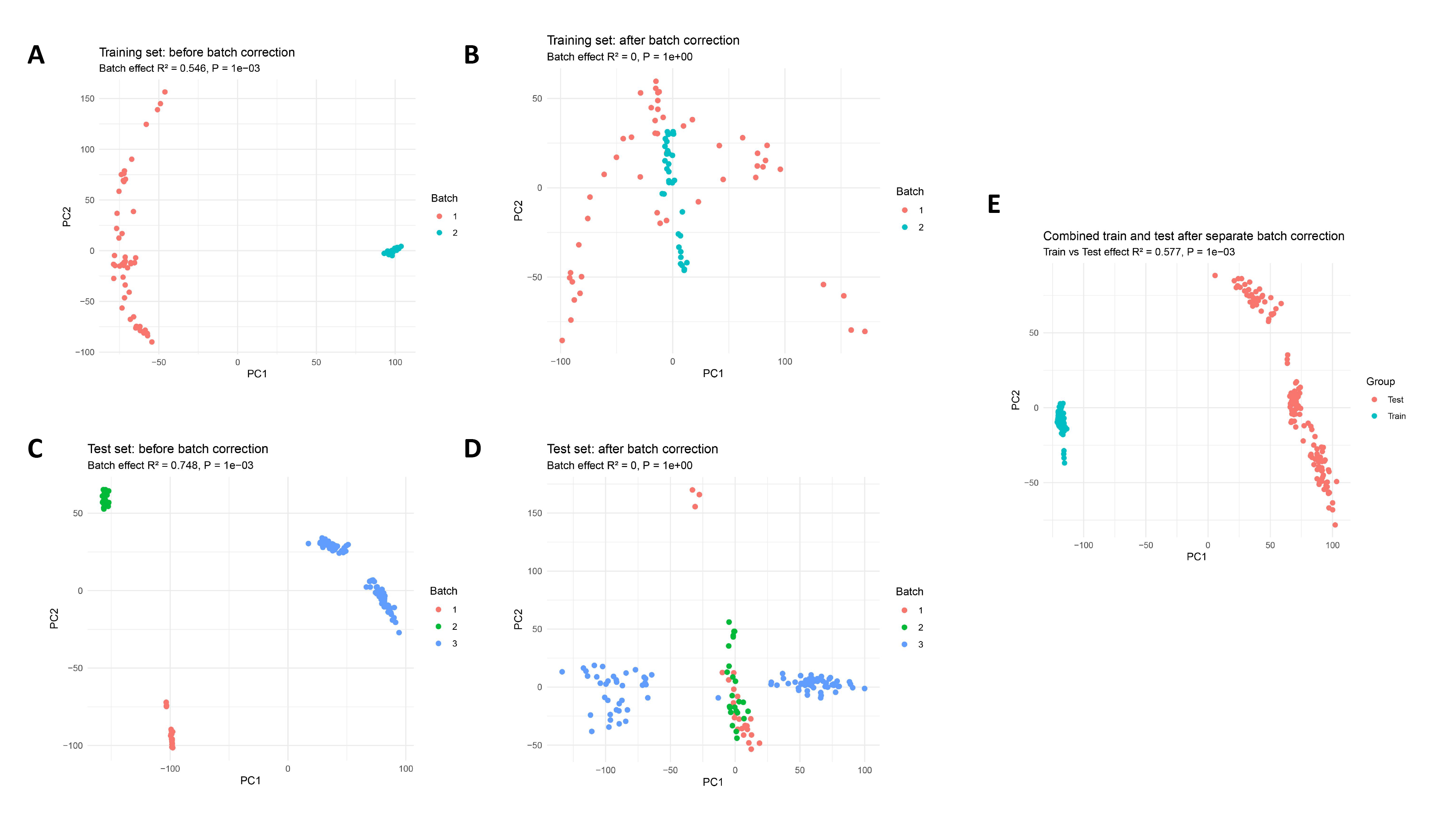

Supplement: Supplementary Figure S1 — PCA of gene expression profiles before and after batch correction. (A) Training set before ComBat correction, points colored by original dataset (batch). (B) Training set after ComBat correction. (C) Test set before correction. (D) Test set after correction. (E) Combined training and test sets after separate batch correction, colored by cohort. The R² and P values shown on each panel are derived from PERMANOVA testing the effect of batch (A–D) or cohort (E). Note that batch effects are almost completely removed within each cohort (R² ≈ 0, P = 1), while a residual biological difference remains between training and test sets (R² = 0.577, P = 0.001), justifying the need for external validation. [file DataSheet1.zip › revised supplementary/Figure S1.tif]

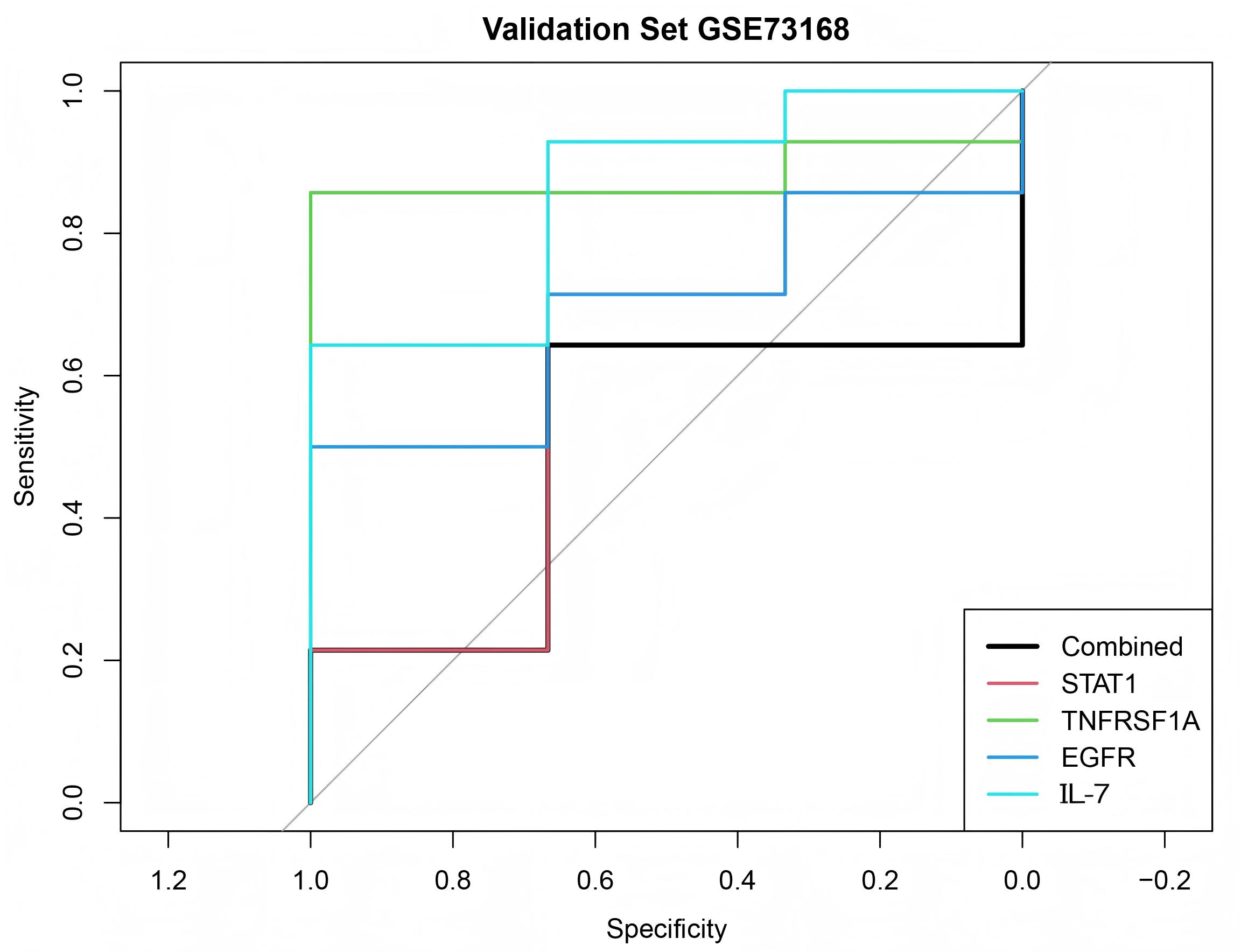

Supplement: Supplementary Figure S1 — PCA of gene expression profiles before and after batch correction. (A) Training set before ComBat correction, points colored by original dataset (batch). (B) Training set after ComBat correction. (C) Test set before correction. (D) Test set after correction. (E) Combined training and test sets after separate batch correction, colored by cohort. The R² and P values shown on each panel are derived from PERMANOVA testing the effect of batch (A–D) or cohort (E). Note that batch effects are almost completely removed within each cohort (R² ≈ 0, P = 1), while a residual biological difference remains between training and test sets (R² = 0.577, P = 0.001), justifying the need for external validation. [file DataSheet1.zip › revised supplementary/Figure S10 ROC_GSE73168.jpg]

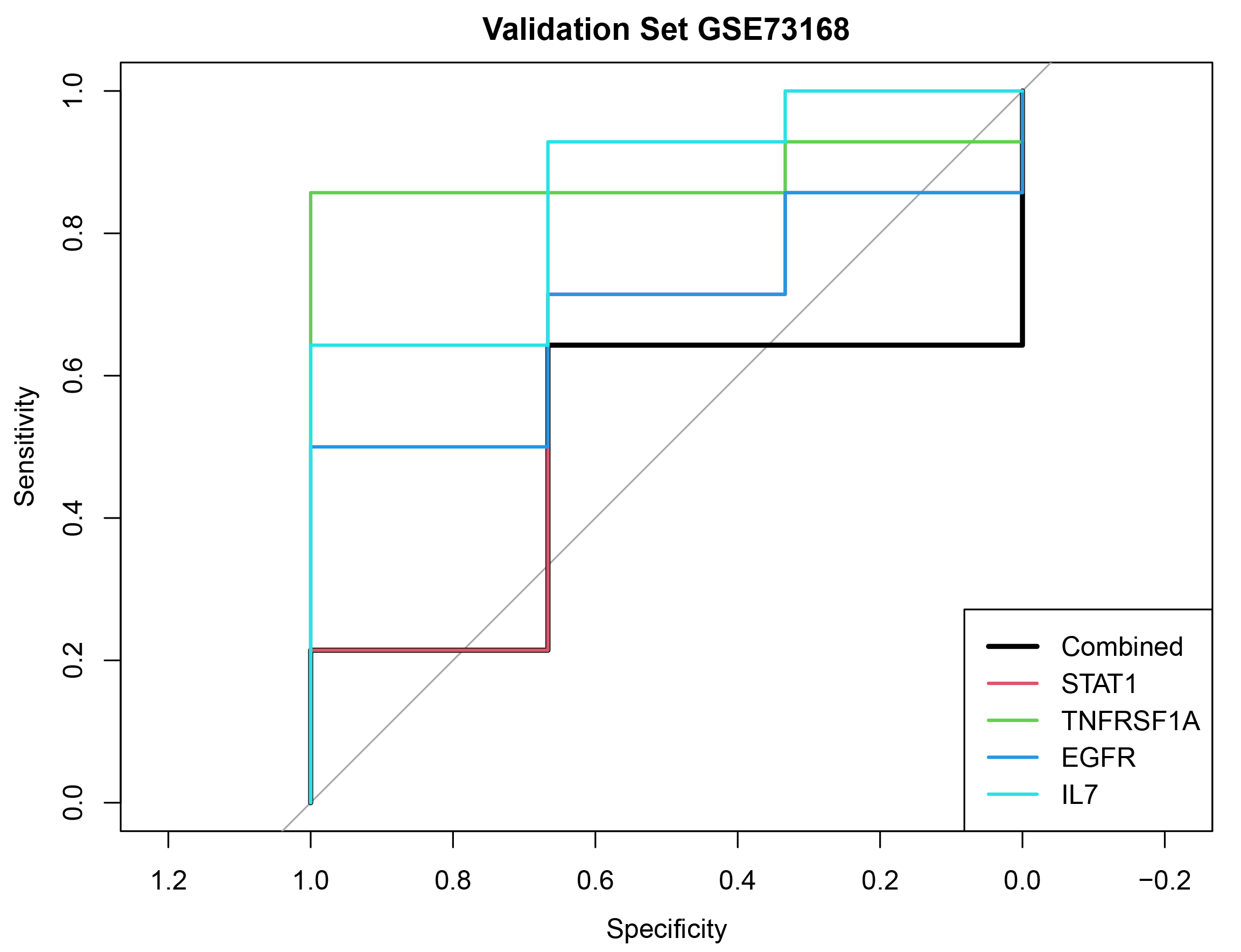

Supplement: Supplementary Figure S1 — PCA of gene expression profiles before and after batch correction. (A) Training set before ComBat correction, points colored by original dataset (batch). (B) Training set after ComBat correction. (C) Test set before correction. (D) Test set after correction. (E) Combined training and test sets after separate batch correction, colored by cohort. The R² and P values shown on each panel are derived from PERMANOVA testing the effect of batch (A–D) or cohort (E). Note that batch effects are almost completely removed within each cohort (R² ≈ 0, P = 1), while a residual biological difference remains between training and test sets (R² = 0.577, P = 0.001), justifying the need for external validation. [file DataSheet1.zip › revised supplementary/Figure S10 ROC_GSE73168.tif]

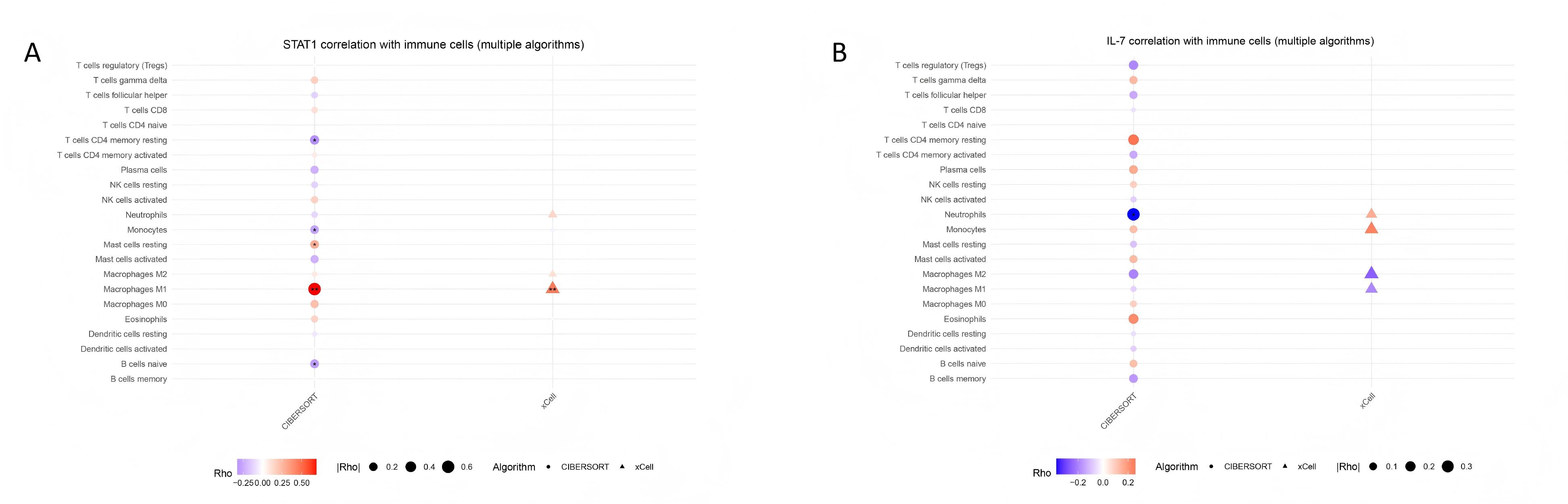

Supplement: Supplementary Figure S1 — PCA of gene expression profiles before and after batch correction. (A) Training set before ComBat correction, points colored by original dataset (batch). (B) Training set after ComBat correction. (C) Test set before correction. (D) Test set after correction. (E) Combined training and test sets after separate batch correction, colored by cohort. The R² and P values shown on each panel are derived from PERMANOVA testing the effect of batch (A–D) or cohort (E). Note that batch effects are almost completely removed within each cohort (R² ≈ 0, P = 1), while a residual biological difference remains between training and test sets (R² = 0.577, P = 0.001), justifying the need for external validation. [file DataSheet1.zip › revised supplementary/Figure S11 Correlation of STAT1 and IL-7 expression with immune cell fractions estimated by CIBERSORT and xCell in HGSOC.jpg]

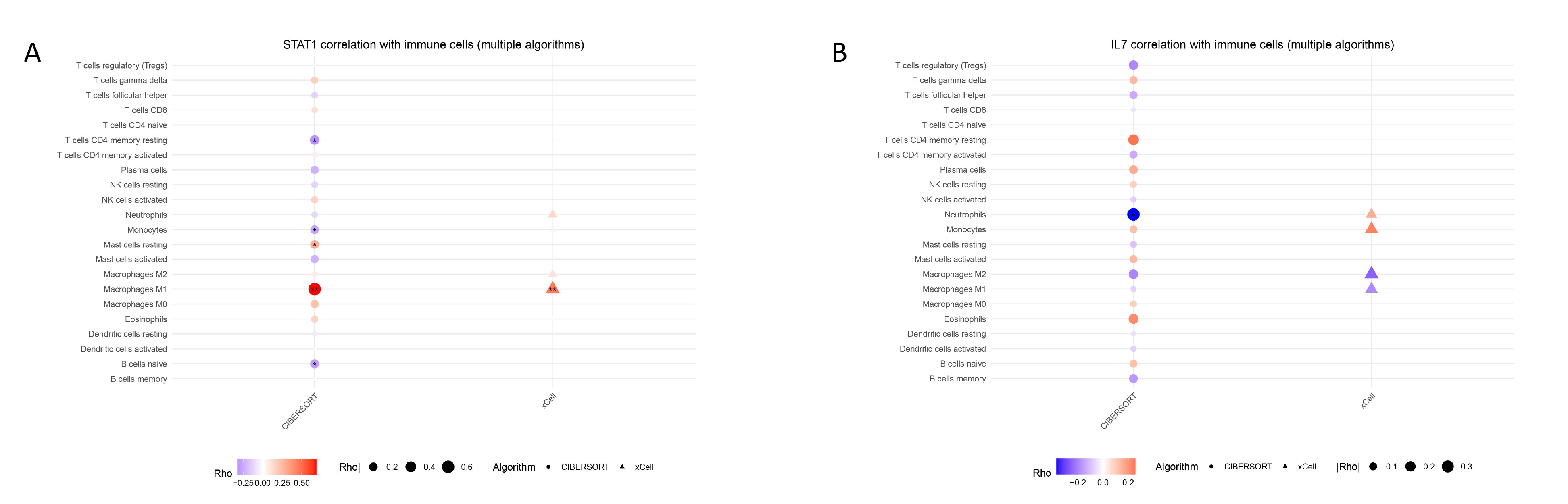

Supplement: Supplementary Figure S1 — PCA of gene expression profiles before and after batch correction. (A) Training set before ComBat correction, points colored by original dataset (batch). (B) Training set after ComBat correction. (C) Test set before correction. (D) Test set after correction. (E) Combined training and test sets after separate batch correction, colored by cohort. The R² and P values shown on each panel are derived from PERMANOVA testing the effect of batch (A–D) or cohort (E). Note that batch effects are almost completely removed within each cohort (R² ≈ 0, P = 1), while a residual biological difference remains between training and test sets (R² = 0.577, P = 0.001), justifying the need for external validation. [file DataSheet1.zip › revised supplementary/Figure S11 Correlation of STAT1 and IL-7 expression with immune cell fractions estimated by CIBERSORT and xCell in HGSOC.tif]

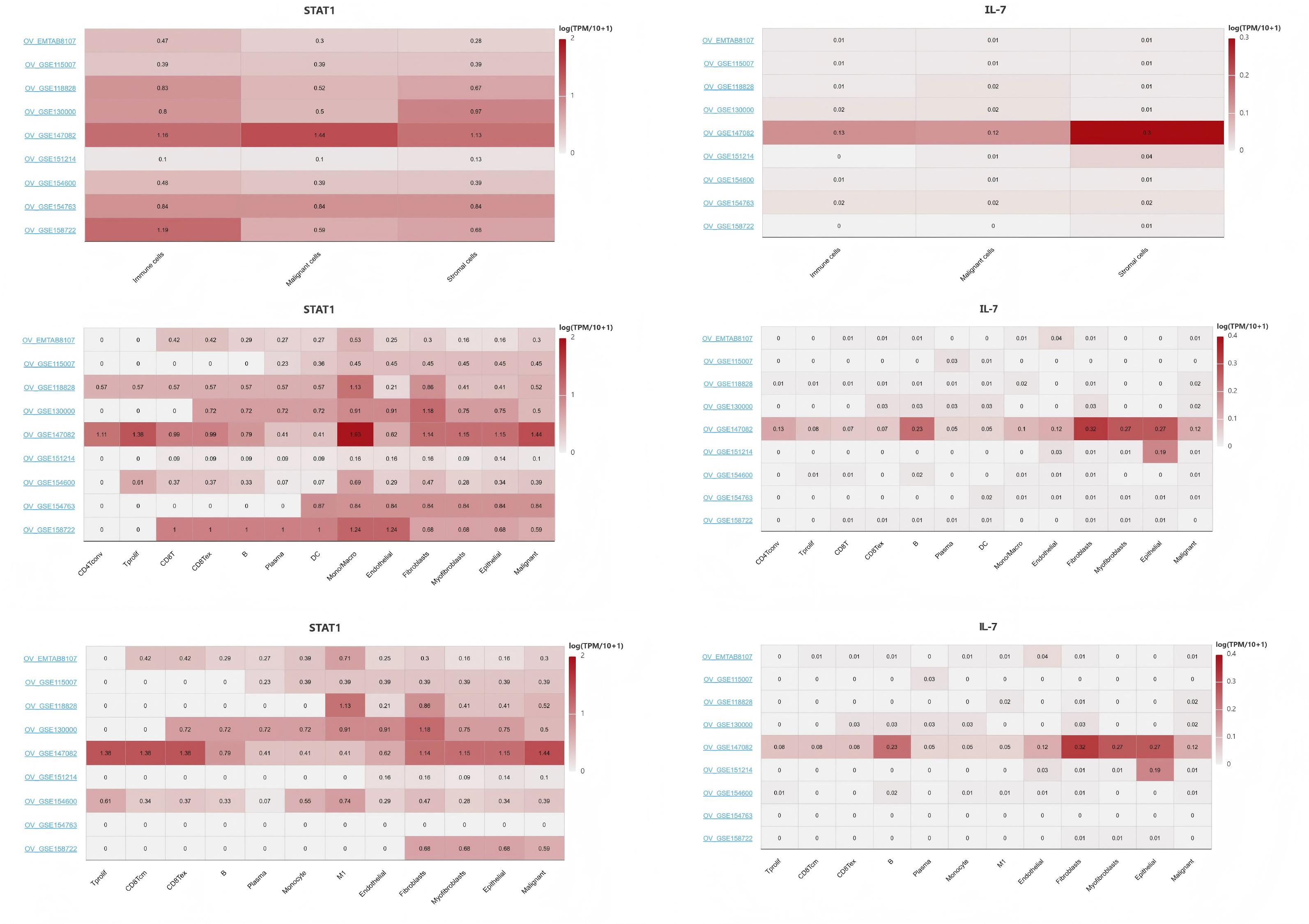

Supplement: Supplementary Figure S1 — PCA of gene expression profiles before and after batch correction. (A) Training set before ComBat correction, points colored by original dataset (batch). (B) Training set after ComBat correction. (C) Test set before correction. (D) Test set after correction. (E) Combined training and test sets after separate batch correction, colored by cohort. The R² and P values shown on each panel are derived from PERMANOVA testing the effect of batch (A–D) or cohort (E). Note that batch effects are almost completely removed within each cohort (R² ≈ 0, P = 1), while a residual biological difference remains between training and test sets (R² = 0.577, P = 0.001), justifying the need for external validation. [file DataSheet1.zip › revised supplementary/Figure S11 STAT1 and IL-7 expression at single-cell resolution using publicly available datasets via the TISCH database .jpg]

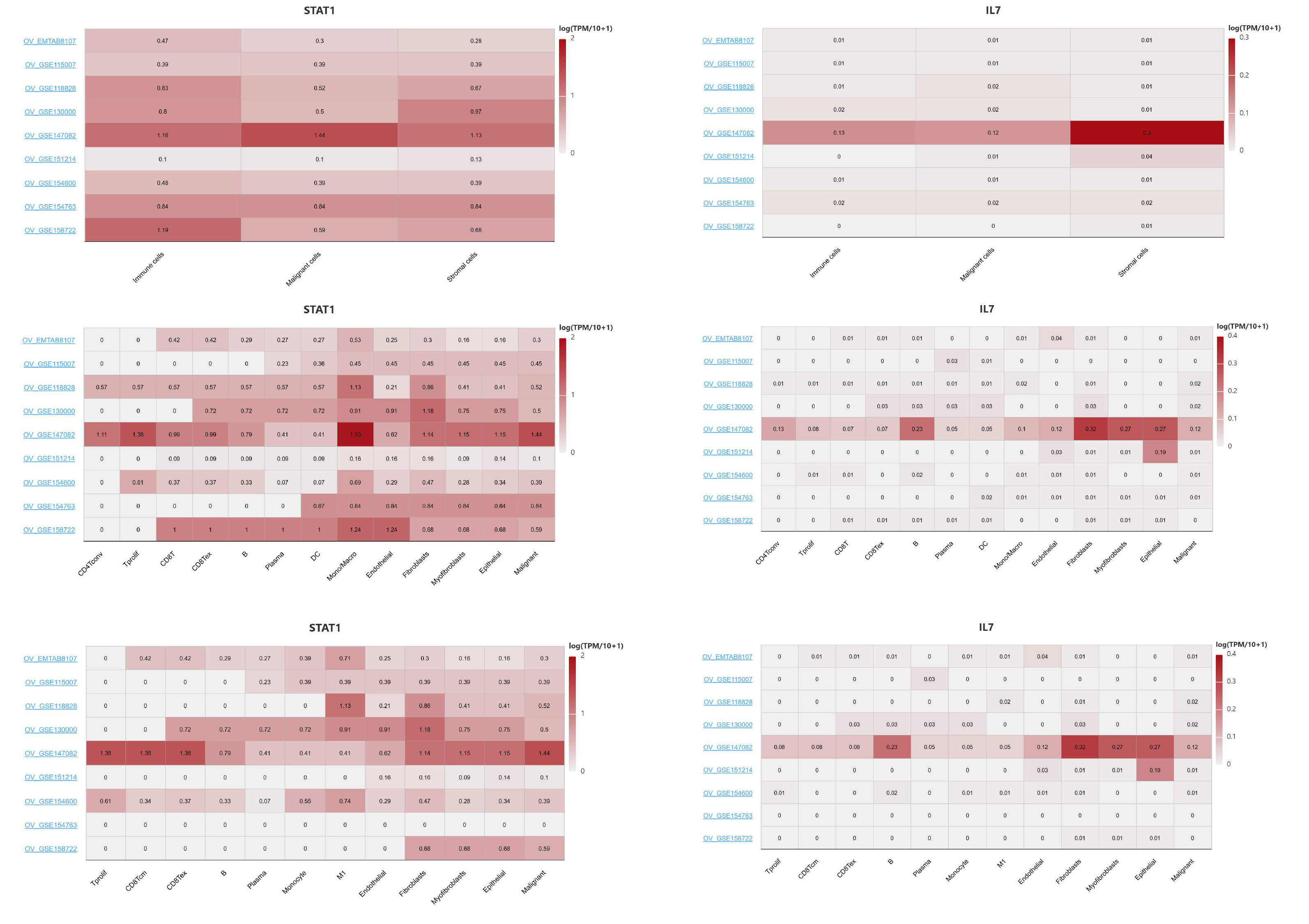

Supplement: Supplementary Figure S1 — PCA of gene expression profiles before and after batch correction. (A) Training set before ComBat correction, points colored by original dataset (batch). (B) Training set after ComBat correction. (C) Test set before correction. (D) Test set after correction. (E) Combined training and test sets after separate batch correction, colored by cohort. The R² and P values shown on each panel are derived from PERMANOVA testing the effect of batch (A–D) or cohort (E). Note that batch effects are almost completely removed within each cohort (R² ≈ 0, P = 1), while a residual biological difference remains between training and test sets (R² = 0.577, P = 0.001), justifying the need for external validation. [file DataSheet1.zip › revised supplementary/Figure S11 STAT1 and IL-7 expression at single-cell resolution using publicly available datasets via the TISCH database .tif]

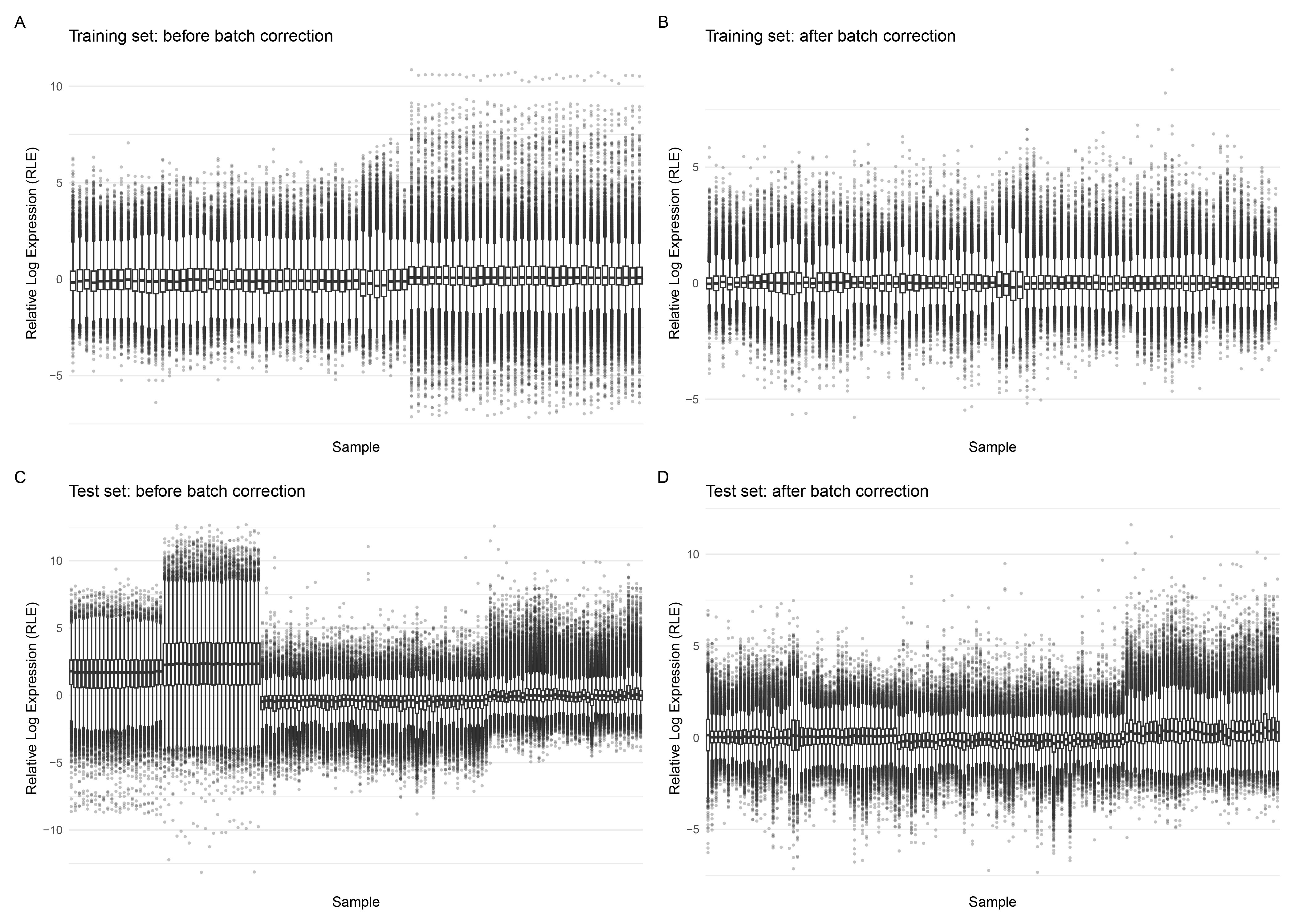

Supplement: Supplementary Figure S1 — PCA of gene expression profiles before and after batch correction. (A) Training set before ComBat correction, points colored by original dataset (batch). (B) Training set after ComBat correction. (C) Test set before correction. (D) Test set after correction. (E) Combined training and test sets after separate batch correction, colored by cohort. The R² and P values shown on each panel are derived from PERMANOVA testing the effect of batch (A–D) or cohort (E). Note that batch effects are almost completely removed within each cohort (R² ≈ 0, P = 1), while a residual biological difference remains between training and test sets (R² = 0.577, P = 0.001), justifying the need for external validation. [file DataSheet1.zip › revised supplementary/Figure S2.tif]

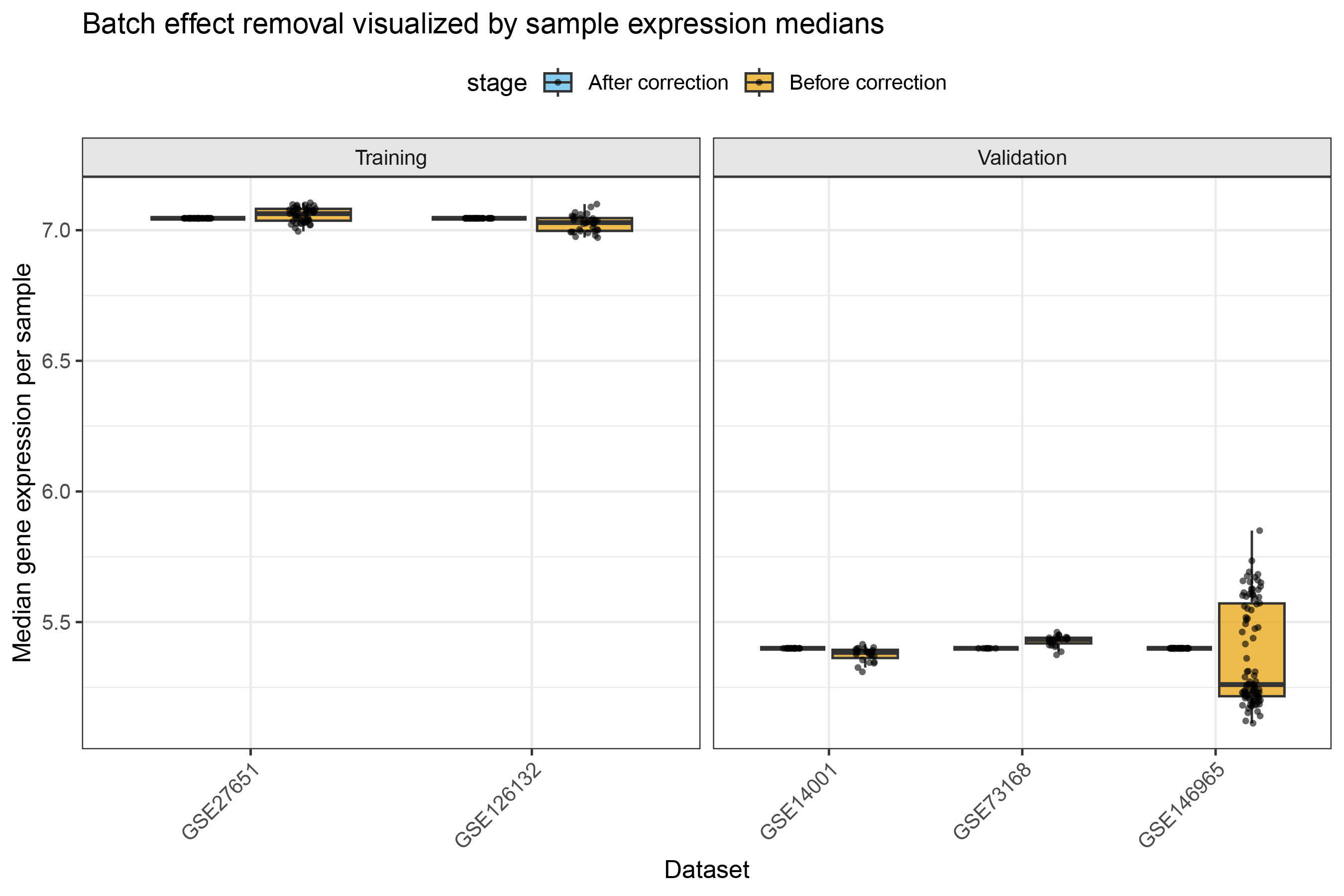

Supplement: Supplementary Figure S1 — PCA of gene expression profiles before and after batch correction. (A) Training set before ComBat correction, points colored by original dataset (batch). (B) Training set after ComBat correction. (C) Test set before correction. (D) Test set after correction. (E) Combined training and test sets after separate batch correction, colored by cohort. The R² and P values shown on each panel are derived from PERMANOVA testing the effect of batch (A–D) or cohort (E). Note that batch effects are almost completely removed within each cohort (R² ≈ 0, P = 1), while a residual biological difference remains between training and test sets (R² = 0.577, P = 0.001), justifying the need for external validation. [file DataSheet1.zip › revised supplementary/Figure S3.tif]

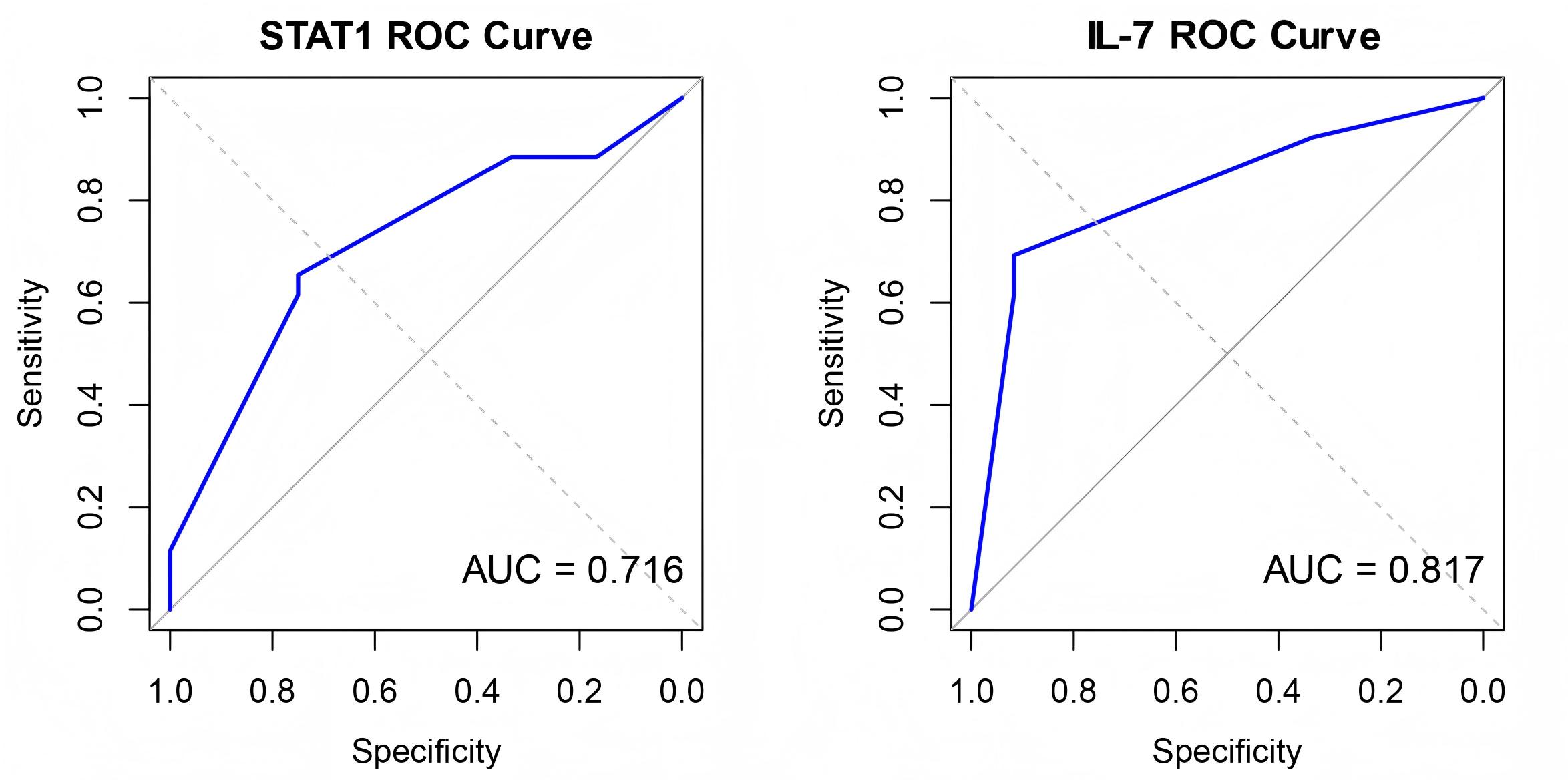

Supplement: Supplementary Figure S1 — PCA of gene expression profiles before and after batch correction. (A) Training set before ComBat correction, points colored by original dataset (batch). (B) Training set after ComBat correction. (C) Test set before correction. (D) Test set after correction. (E) Combined training and test sets after separate batch correction, colored by cohort. The R² and P values shown on each panel are derived from PERMANOVA testing the effect of batch (A–D) or cohort (E). Note that batch effects are almost completely removed within each cohort (R² ≈ 0, P = 1), while a residual biological difference remains between training and test sets (R² = 0.577, P = 0.001), justifying the need for external validation. [file DataSheet1.zip › revised supplementary/Figure S4 ROC_curves(IHC).jpg]

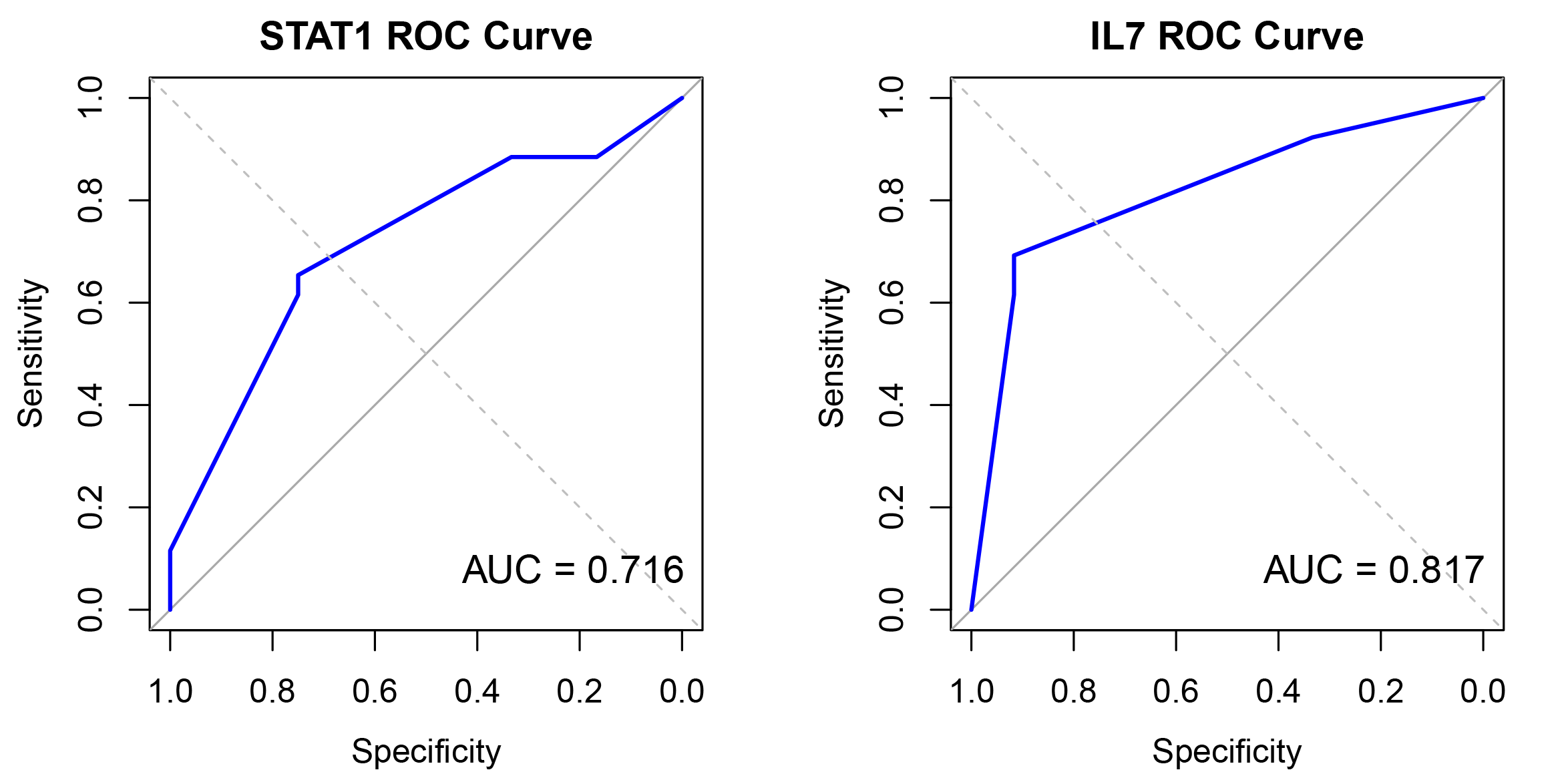

Supplement: Supplementary Figure S1 — PCA of gene expression profiles before and after batch correction. (A) Training set before ComBat correction, points colored by original dataset (batch). (B) Training set after ComBat correction. (C) Test set before correction. (D) Test set after correction. (E) Combined training and test sets after separate batch correction, colored by cohort. The R² and P values shown on each panel are derived from PERMANOVA testing the effect of batch (A–D) or cohort (E). Note that batch effects are almost completely removed within each cohort (R² ≈ 0, P = 1), while a residual biological difference remains between training and test sets (R² = 0.577, P = 0.001), justifying the need for external validation. [file DataSheet1.zip › revised supplementary/Figure S4 ROC_curves(IHC).tif]

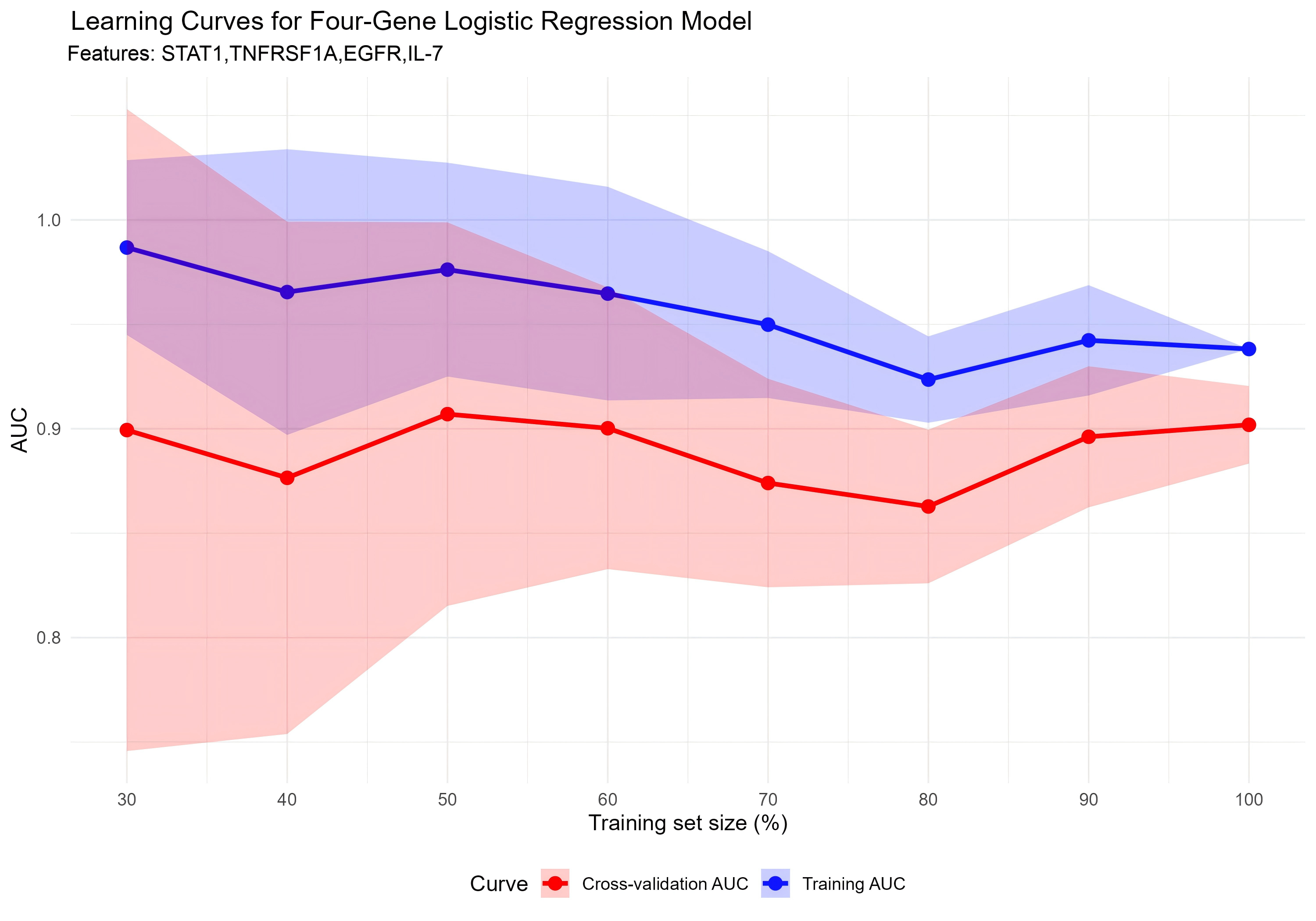

Supplement: Supplementary Figure S1 — PCA of gene expression profiles before and after batch correction. (A) Training set before ComBat correction, points colored by original dataset (batch). (B) Training set after ComBat correction. (C) Test set before correction. (D) Test set after correction. (E) Combined training and test sets after separate batch correction, colored by cohort. The R² and P values shown on each panel are derived from PERMANOVA testing the effect of batch (A–D) or cohort (E). Note that batch effects are almost completely removed within each cohort (R² ≈ 0, P = 1), while a residual biological difference remains between training and test sets (R² = 0.577, P = 0.001), justifying the need for external validation. [file DataSheet1.zip › revised supplementary/Figure S5 LearningCurves.png]

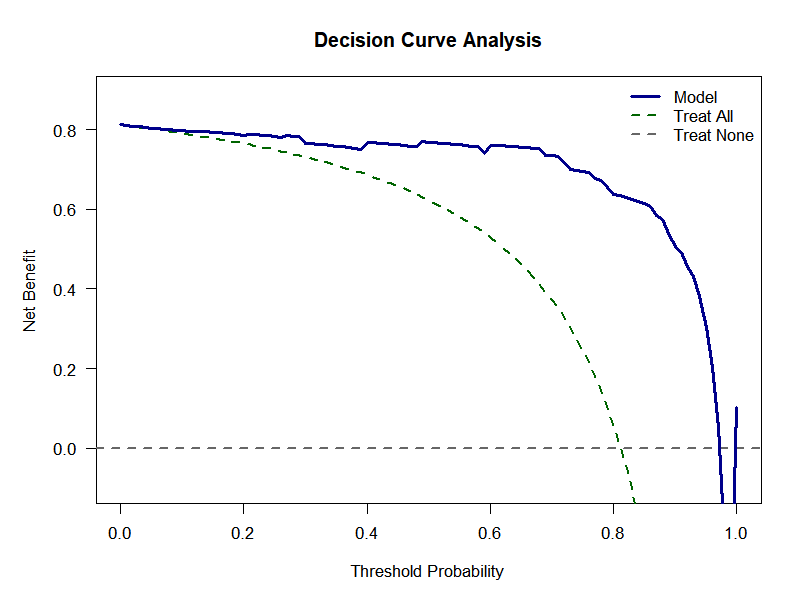

Supplement: Supplementary Figure S1 — PCA of gene expression profiles before and after batch correction. (A) Training set before ComBat correction, points colored by original dataset (batch). (B) Training set after ComBat correction. (C) Test set before correction. (D) Test set after correction. (E) Combined training and test sets after separate batch correction, colored by cohort. The R² and P values shown on each panel are derived from PERMANOVA testing the effect of batch (A–D) or cohort (E). Note that batch effects are almost completely removed within each cohort (R² ≈ 0, P = 1), while a residual biological difference remains between training and test sets (R² = 0.577, P = 0.001), justifying the need for external validation. [file DataSheet1.zip › revised supplementary/Figure S6 DCA.png]

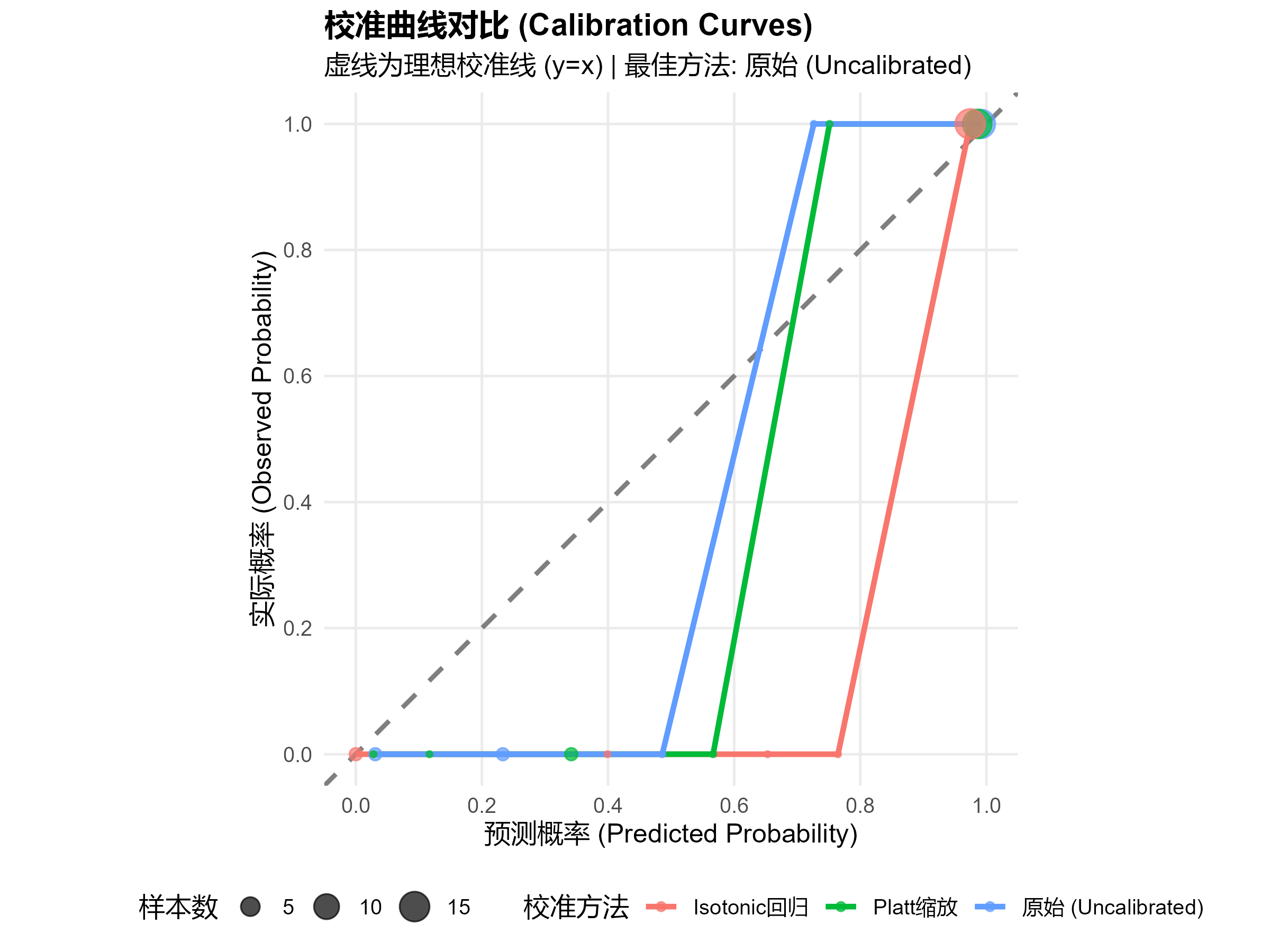

Supplement: Supplementary Figure S1 — PCA of gene expression profiles before and after batch correction. (A) Training set before ComBat correction, points colored by original dataset (batch). (B) Training set after ComBat correction. (C) Test set before correction. (D) Test set after correction. (E) Combined training and test sets after separate batch correction, colored by cohort. The R² and P values shown on each panel are derived from PERMANOVA testing the effect of batch (A–D) or cohort (E). Note that batch effects are almost completely removed within each cohort (R² ≈ 0, P = 1), while a residual biological difference remains between training and test sets (R² = 0.577, P = 0.001), justifying the need for external validation. [file DataSheet1.zip › revised supplementary/Figure S7 Calibration curve comparison demonstrating that the original two-gene logistic regression model.png]

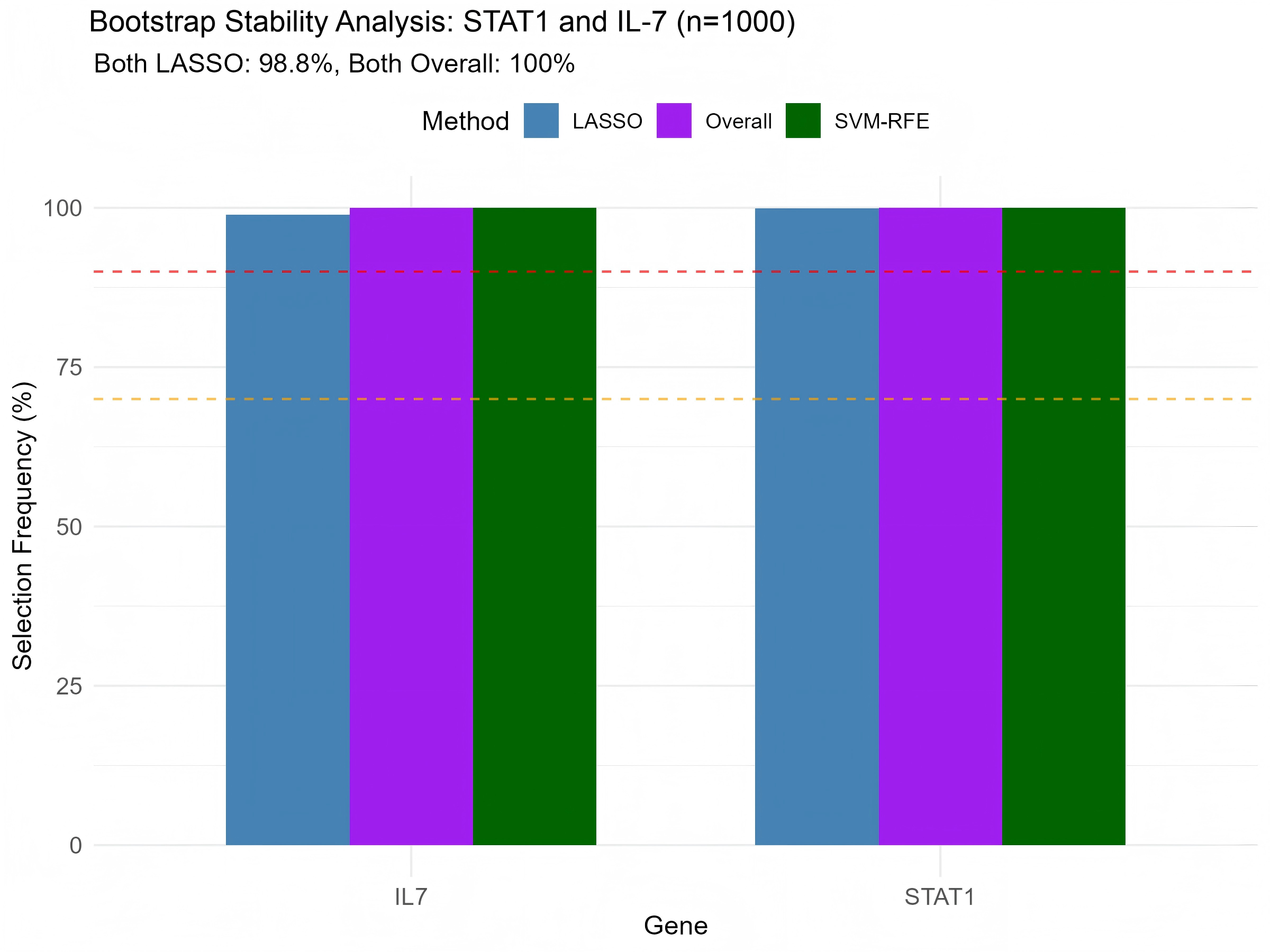

Supplement: Supplementary Figure S1 — PCA of gene expression profiles before and after batch correction. (A) Training set before ComBat correction, points colored by original dataset (batch). (B) Training set after ComBat correction. (C) Test set before correction. (D) Test set after correction. (E) Combined training and test sets after separate batch correction, colored by cohort. The R² and P values shown on each panel are derived from PERMANOVA testing the effect of batch (A–D) or cohort (E). Note that batch effects are almost completely removed within each cohort (R² ≈ 0, P = 1), while a residual biological difference remains between training and test sets (R² = 0.577, P = 0.001), justifying the need for external validation. [file DataSheet1.zip › revised supplementary/Figure S8 Bootstrap Stability Analysis STAT1 and IL-7 (n=1oo0).png]

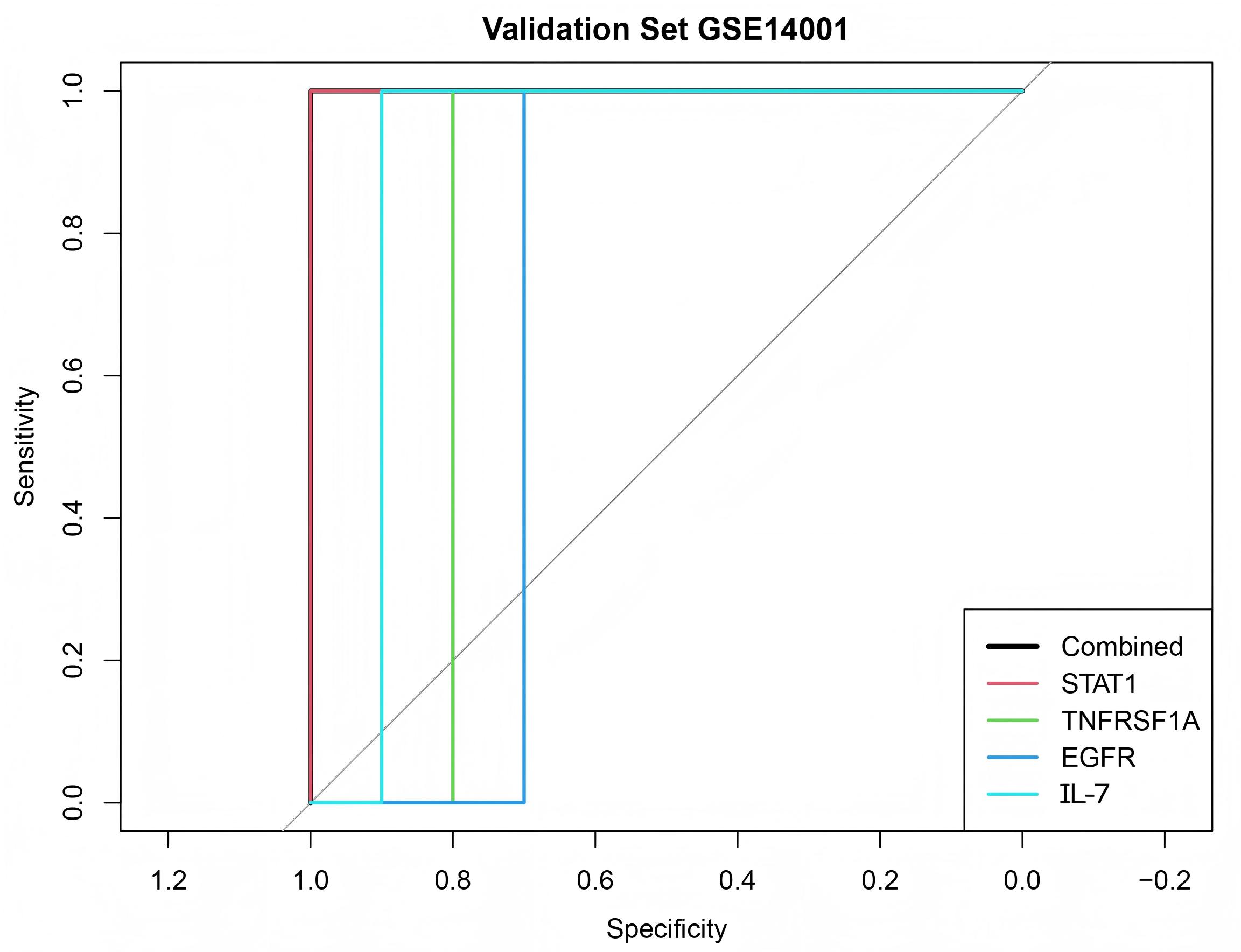

Supplement: Supplementary Figure S1 — PCA of gene expression profiles before and after batch correction. (A) Training set before ComBat correction, points colored by original dataset (batch). (B) Training set after ComBat correction. (C) Test set before correction. (D) Test set after correction. (E) Combined training and test sets after separate batch correction, colored by cohort. The R² and P values shown on each panel are derived from PERMANOVA testing the effect of batch (A–D) or cohort (E). Note that batch effects are almost completely removed within each cohort (R² ≈ 0, P = 1), while a residual biological difference remains between training and test sets (R² = 0.577, P = 0.001), justifying the need for external validation. [file DataSheet1.zip › revised supplementary/Figure S9 ROC_GSE14001.jpg]

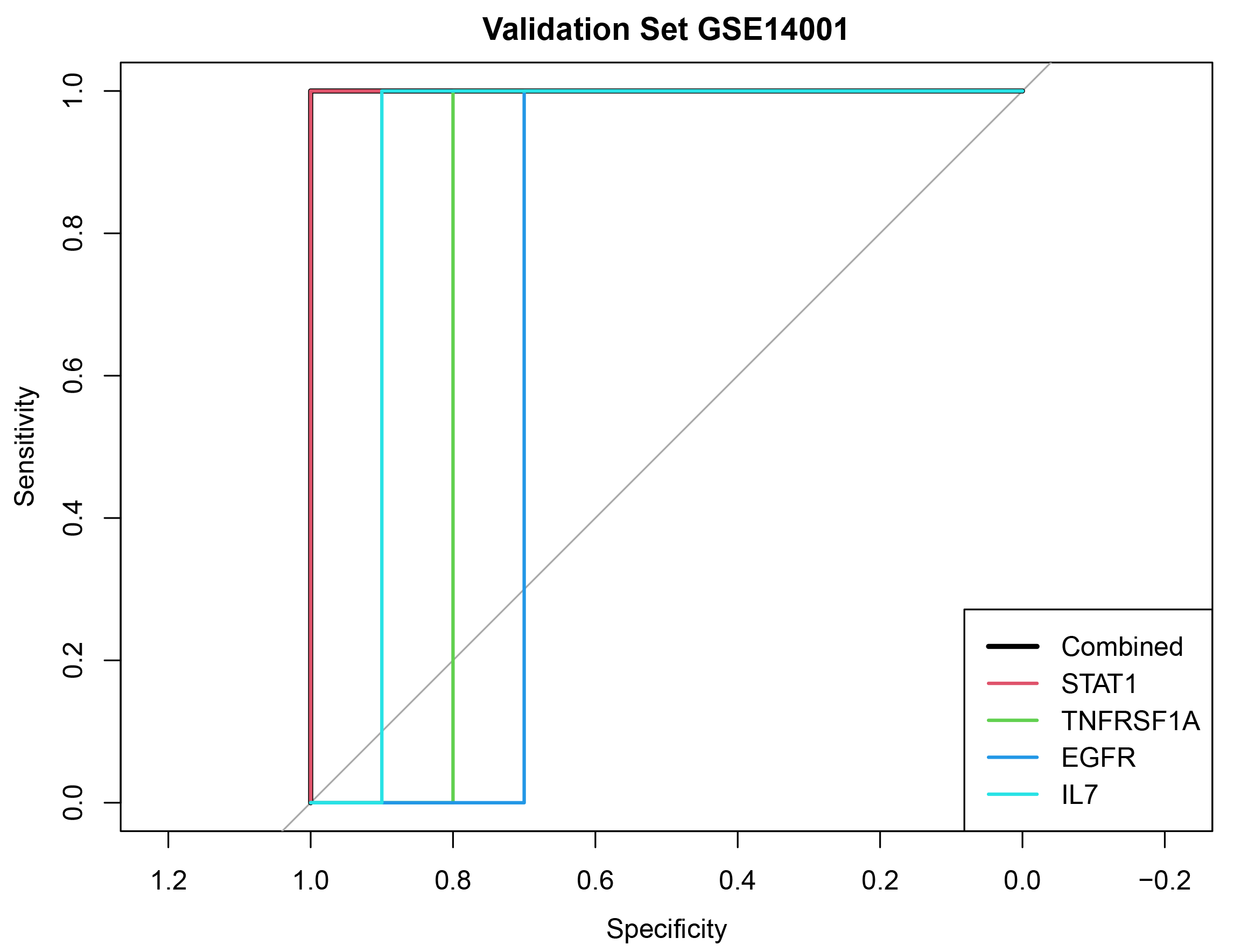

Supplement: Supplementary Figure S1 — PCA of gene expression profiles before and after batch correction. (A) Training set before ComBat correction, points colored by original dataset (batch). (B) Training set after ComBat correction. (C) Test set before correction. (D) Test set after correction. (E) Combined training and test sets after separate batch correction, colored by cohort. The R² and P values shown on each panel are derived from PERMANOVA testing the effect of batch (A–D) or cohort (E). Note that batch effects are almost completely removed within each cohort (R² ≈ 0, P = 1), while a residual biological difference remains between training and test sets (R² = 0.577, P = 0.001), justifying the need for external validation. [file DataSheet1.zip › revised supplementary/Figure S9 ROC_GSE14001.tif]
