# Supplementary material for: STAT1 and IL-7 as potential diagnostic biomarkers for distinguishing high-grade from low-grade serous ovarian cancer: a multi-cohort analysis
Source: Front Immunol. 2026 Apr 14;17:1779912. doi: 10.3389/fimmu.2026.1779912 (PMC13120972; doi:10.3389/fimmu.2026.1779912)
Supplement: Supplementary Table S1 — PERMANOVA results quantifying batch effects before and after ComBat correction. [file Table1.doc]

<https://www.jianguoyun.com/c/sd/1c2ac68/6dd9c4f4c57280f2>

<https://www.jianguoyun.com/c/sd/1c2ac68/6dd9c4f4c57280f2>
